# Transcriptome of Cydia pomonella granulovirus in susceptible and type I resistant codling moth larvae

**DOI:** 10.1099/jgv.0.001566

**Published:** 2021-02-24

**Authors:** Jörg T. Wennmann, Diana Pietruska, Johannes A. Jehle

**Affiliations:** ^1^​ Julius Kühn Institute (JKI) – Federal Research Centre for Cultivated Plants, Institute for Biological Control, Heinrichstr. 243, 64287 Darmstadt, Germany

**Keywords:** baculovirus, CpGV, Cydia pomonella, gene expression, microarray, resistance, RNA, virus replication

## Abstract

The baculovirus Cydia pomonella granulovirus (CpGV) is a biocontrol agent used worldwide against the codling moth (CM), *Cydia pomonella* L., a severe pest in organic and integrated pome fruit production. Its successful application is increasingly challenged by the occurrence of CM populations resistant to commercial CpGV products. Whereas three types (I–III) of CpGV resistance have been identified, type I resistance compromising the efficacy of CpGV-M, the so-called Mexican isolate of CpGV, is assumed to be the most widely distributed resistance type in Central Europe. Despite the wide use of CpGV products as biocontrol agents, little information is available on gene-expression levels in CM larvae. In this study, the *in vivo* transcriptome of CpGV-M infecting susceptible (CpS) and resistant (CpRR1) CM larvae was analysed at 24, 48, 72, 96 and 120 hours post infection in the midgut and fat body tissue by using a newly developed microarray covering all ORFs of the CpGV genome. According to their transcript abundance, the CpGV genes were grouped into four temporal clusters to which groups of known and unknown function could be assigned. In addition, sets of genes differentially expressed in the midgut and fat body were found in infected susceptible CpS larvae. For the resistant CpRR1 larvae treated with CpGV-M, viral entry in midgut cells could be confirmed from onset but a significantly reduced gene expression, indicating that type I resistance is associated with a block of viral gene transcription and replication.

## Introduction

The Cydia pomonella granulovirus of the family *Baculoviridae*, genus *Betabaculovirus,* is a dsDNA virus with a covalently closed genome of 120.8 to 124.3 kbp encoding up to 142 ORFs [1, 2]. It is highly infectious for larval instars of the codling moth (CM), *Cydia pomonella* (L.), which is a serious pest with a nearly worldwide distribution in apple-growing regions. Due to its high virulence and narrow host range to CM larvae and its harmlessness towards non-target organisms and the environment [3, 4], CpGV became one of the most widely applied and commercially important baculoviruses and serves as a cornerstone of biological CM control in integrated and organic apple and pear production [5, 6]. The first CpGV isolate was discovered in Mexico (CpGV-M) [7] and it has been used successfully in commercial biocontrol products since the late 1980s. Today many different geographic field isolates are known [8–12]. These isolates show different levels of virulence and can be divided into seven phylogenetic genome groups, termed A to G, according to comparative genome studies [1, 2].

Based on quantitative analyses of single nucleotide polymorphisms (SNPs), it was possible to identify genotypically homogeneous and heterogeneous, and mixed isolates [1, 13, 14]. In addition to natural field isolates, commercially selected isolates of genome groups A, B and E are also known [5, 6, 15–17], some being mixtures of these genome groups [16].

In 2005, the first emergence of CM field populations resistant to commercial products containing CpGV-M (genome group A) was documented in Germany and France [18, 19] and later in other European countries [20–24]. This first case of field resistance to commercial baculovirus products was reflected by a 1000- to 100 000-fold reduced susceptibility of CM larvae to CpGV-M (genome group A) and is referred to as type I resistance [25]. The nature of this type I resistance was better understood following generation of the laboratory CM strain CpRR1, a genetically homogeneous inbred strain that originated from a resistant field population in southern Germany. In CpRR1, CpGV resistance is inherited dominantly on the sex-chromosome (Z chromosome) [23, 25]. Today two other types of CpGV resistance are known (type II and type III resistance), which are directed against other CpGV isolates and exhibit different inheritance patterns [26–28].

For type I resistance, the ORF *pe38* was identified as a resistance-related viral genetic factor by comparing the genome sequences of CpGV-M, and the resistance-breaking English isolate CpGV-E2 (genome group B), the Iranian isolates CpGV-I07 and CpGV-I12 (genome groups C and D, respectively), as well as CpGV-S (genome group E) [29]. The common difference between all these resistance-breaking CpGV isolates and CpGV-M was an additional 2×12 bp long repeat motif in *pe38* of CpGV-M, where this 12 bp repeat motif occurs three times [1, 13, 29]. Resistance-breaking isolates, such as CpGV-S, CpGV-E2 and CpGV-I12, are characterized by genotypes with a single 12 bp repeat motif [8, 13, 29]. The genotypic frequency of a single 12 bp repeat motif within an isolate correlates with its resistance-breaking activity in CpRR1 [8, 13, 16, 27, 28]. To date, the function of *pe38* in CpGV remains unknown although its gene sequence indicates a zinc finger and leucine zipper domain in its protein. In addition to its function, the temporal expression and level of transcription of *pe38* are still unknown.

With the help of a genetically modified CpGV-M bacmid encoding for an enhanced green fluorescent protein (eGFP), it was demonstrated that CpGV-M is able to enter resistant CpRR1 cells but its viral replication is blocked [30]. Although the genome sequences of more than 20 geographic and commercial CpGV isolates have been extensively investigated, very little is known about gene transcription of CpGV. One reason is the lack of a highly permissive cell line; hence expression studies are restricted to asynchronous infections of CM host larvae and their tissues. An example of a global transcription analysis under conditions in cell culture or larval midguts is the Autographa californica nucleopolyhedrovirus (AcMNPV), belonging to the genus *Alphabaculovirus* [31, 32].

In the present work, the global transcription of a permissive infection of CpGV-M in susceptible CM larvae (CpS) was measured for the midgut and fat body tissue at different time points. The results were compared with the onset of gene expression of CpGV-M in the midgut of resistant CpRR1. Our findings make an important contribution to the understanding of virus–host interaction, the transcriptome of CpGV and baculoviruses in general.

## Methods

### Insects and viruses

Laboratory strains of susceptible (CpS) and type I resistant (CpRR1) *C. pomonella* larvae were maintained at the Julius Kühn-Institut, Institute for Biological Control, in Darmstadt, Germany. Both CM strains were reared under the same laboratory conditions [33]. Neonate larvae were either used directly for experimental purposes or kept on semi-artificial diet [34] until they reached the desired larval stage.

Two isolates of the Cydia pomonella granulovirus (CpGV) were used in this study: the Mexican isolate (CpGV-M) [7] and the Iranian isolate (CpGV-I12) [11]. Both isolates were initially propagated in susceptible fourth instar CpS larvae. The protocol for occlusion-body (OB) purification followed the method described by Jehle *et al*. [35]. Stocks of OB suspensions of CpGV-M and CpGV-I12 were stored at −20 °C and were enumerated using a Petroff-Hauser counting chamber (depth 0.02 mm) in dark-field optics of a Leica light microscope (DMRBE) [36].

### Infection experiments for transcriptome analysis

Batches of CpS and CpRR1 neonate larvae were reared individually on artificial diet [34] and were checked daily for signs of moulting, such as lost head capsules. After approximately, 4 to 5 days, larvae underwent the third larval stage. Larvae of the same size and that reached the fourth larval stage on the same day were considered to be at the same stage of development and used for the infection experiments. Larvae were starved individually overnight and were fed subsequently with a small cube (1 mm^3^) of semi-artificial diet containing 10^3^ OBs of either CpGV-M or CpGV-I12. Control larvae were provided diet without virus OBs. After 4 h, larvae were checked if they had eaten the entire piece of diet. Larvae that did not entirely ingest the provided inoculum were excluded from all further analyses. All successfully inoculated larvae were then kept on virus-free diet and this time point was set as the experimental starting point (=zero h post-infection, h p.i.). At time points 0, 12, 24, 48, 72, 96 and 120 h p.i. a subset of 30 to 50 larvae was removed from the experiment. Larvae fed on virus-free control diet (untreated control) were collected at 120 h p.i. Larval samples from different time points were stored individually in a 1.5 ml centrifuge tube at −80 °C until midgut and fat body dissection and RNA isolation. The entire experimental setup was repeated independently three times.

### Tissue dissection and RNA isolation

Dissection of midgut and fat body tissues was performed for a randomly chosen subset of ten larvae of each replicate and time point. Tissue preparation was done rapidly under a binocular on ice-cooled glass Petri dishes to avoid any unnecessary damage to larval tissues and degradation of RNA. Midguts were washed to remove intestinal debris. Due to the small size of the larvae, the fat body of the entire larvae was included in the dissection process without specifying its association to other larval organs. Midgut and fat body tissues of all ten larvae were separately pooled in lysis buffer of the RNA purification kit (GeneJET, Thermo Fisher) containing β-mercaptoethanol (BME). Isolation of total RNA was performed immediately after the dissection process on each set of pooled midguts or fat body tissue following the manufacturer’s manual of the RNA purification kit. Briefly, the pooled tissues were homogenized within a 1.5 ml centrifuge tube using a micro pestle. The homogenate was treated with proteinase K at a final concentration of 250 µg ml^−1^ at 25 °C for 10 min and centrifuged at 12 000 r.c.f. for 5 min. Supernatants were transferred to RNase-free centrifuge tubes and mixed with 96 % ethanol. After careful mixing by inversion, the RNA containing mixture was centrifuged through a RNA binding column. The column was washed twice with washing buffers I and II supplied with the RNA purification kit followed by centrifugation at 12 000 r.c.f. for 1 min and 2 min, respectively. Finally, the RNA was eluted from the column by adding 50 µl RNAase-free water and by centrifugation at 12 000 r.c.f. for 1 min.

### RNA quality control

Prior to the DNAase I treatment the amounts of RNA in the samples were measured using a eukaryote total RNA nano kit (Agilent) in a Bioanalyzer 2100 Expert (Agilent) and checked for *C. pomonella*-specific RNA chromatograms. From the chromatograms the level and RNA degradation were evaluated by the help of a 6000 nt (6000, 4000, 2000, 1000, 500, 200 and 25 nt) nano marker. The RNA quality was estimated by the ratio of 18S and 28S rRNA to calculate the RNA integrity number (RIN) ranging from 10 (optimal) to 1 (entirely degraded). Samples with a RIN >8 were considered as sufficient for the microarray study. In cases where the larval RNA samples appeared degraded, the larval sample was replaced with a new one following the above described tissue dissection and RNA isolation protocol. An entity of ten high-quality RNA samples were pooled and prepared for the microarray studies. Three biological replicates for each time point were generated.

### DNAse treatment

To remove any DNA contamination, 5 µl (1 U µl^−1^) DNAase I and 5 µl DNAse I buffer were added to each 50 µl RNA sample followed by incubation at 37 °C for 30 min. DNAse I was then inactivated by adding 5 µl EDTA (50 mM) and incubation at 65 °C for 10 min. Eventually, the RNA samples were checked for DNA contamination by PCR analysis using *actin* gene specific primers: 5′-AGTACGTACGTGTTGGCCATG-3′ (actin forward primer) and 5′-AGTACGTACGTGTTGGCCATG-3′ (actin reverse primer). For a PCR, 2 µl of RNA sample were mixed with 1 µl 10 µM of each forward and reverse primer, respectively, 5 µl 10× reaction buffer (Axon), 4 µl 25 mM MgCl_2_, 1 µl of a mixture of 10 mM (each) dNTP, 0.5 µl (5 U µl^−1^) *Taq* polymerase (Axon) and 35.5 µl ddH_2_O to a final volume of 50 µl. PCR reactions were initiated by DNA denaturation for at 94 °C for 1 min, followed by 35 cycles of denaturation at 94 °C for 1 min, primer annealing at 50 °C for 45 s and elongation at 72 °C for 1 min. Final elongation was performed at 72 °C for 5 min. Genomic DNA of *C. pomonella* was used for positive- and water for negative-control reactions. PCR fragments were separated by gel electrophoresis and staining of DNA by Midori Green Advance (Nippon Genetics Europe GmbH, Düren, Germany).

### Reverse transcription of RNA

Synthesis of cDNA was performed for RNA samples of the untreated control and of the treatments at 0, 12, 24, 48, 72, 96 and 120 h p.i. using the iScript cDNA synthesis kit (BioRad) and following the manufacturer’s protocol. For a reverse transcription reaction 22 µl of nucleotide free H_2_O, 8 µl reaction buffer, 2 µl iScript reverse transcriptase (RT) and 2 µl RNA sample (50 ng µl^−1^) were mixed. RT control reactions were set up with 2 µl of nucleotide-free H_2_O instead of the RNA sample in order to check for RNA contamination. Priming using the kit’s random hexamers was performed at 25 °C for 5 min, followed by reverse transcription at 46 °C for 20 min and RT inactivation at 95 °C for 1 min.

### Quantitative PCR (qPCR)-based transcriptome analysis

Levels of gene expression of viral genes *ie-1, pe38, lef-8, f-protein, vp39* and *granulin* (*gran*) as well as the house-keeping gene *actin* were determined by qPCR analysis using a CFX96 real-time system (Bio-Rad, Hercules, CA, USA). Each reaction was performed in 25 µl total volume with 1 µl (30 µM) of each forward and reverse primer (Table 1), 12.5 µl Maxima SYBR Green/Rox qPCR master mix (Thermo Fisher Scientific, Waltham, MA, USA), 2 µl cDNA and 8.5 µl ddH_2_O. The program was set as follows: initial denaturation at 95 °C for 2 min followed by 45 cycles of denaturation at 94 °C for 1 min, annealing at 60 °C for 30 s and elongation at 72 °C for 30 s. After completion of all qPCR cycles, melting curve analysis was performed from 60–95 °C with an increment of 0.5 °C each 5 s. Non-target controls, non-RT and negative RT samples were included in each quantification experiment. Data analysis was conducted with Bio-Rad CFX Manager 3.0 (Bio-Rad, Hercules, CA, USA) to obtain Cq (quantification cycle) data. Cq values of each biological replicate of all time points (0, 12, 24, 48, 72, 96 and 120 h p.i.) were measured by three technical replicates. The average Cq value of all three replicates was normalized by the average Cq value of the house-keeping gene *actin*. The relative gene expression was calculated by 2^-∆Ct^.

**Table 1.** Oligonucleotides used in RT-qPCR for analysing the gene-expression level of selected early, late and very late genes. Quantitation of viral gene expression was determined relative to the expression level of the host house-keeping gene actin

Name	Target gene	Expression level	Sequence (5′ to 3′)	Product size (bp)
pCM_actin_f	*actin*	house-keeping gene	AATGGCTCCGGTATGTGC	216
pCM_actin_r			TTGCTCTGT GCCTCGTCT	
pCpGV-M_ie-1_f	*ie-1*	early	CCCCAATCCTATGAGAAGCA	221
pCpGV-M_ie-1_r			ACGCTTTCGAAATGACCATC	
pCpGV-M_lef-8_f	*lef-8*	early	CTTCCGTCTTCAACCTACTGT	452
pCpGV-M_lef-8_r			CGCGCCCGTGGTGATAAAAC	
pCpGV-M_pe38_f	*pe38*	early	CACGAAGCAGCACTCATTGT	181
pCpGV-M_pe38_r			GCGGTGCTTTAACAGTCCTC	
pCpGV-M_f-protein_f	*f-protein*	late	GACAGGGACGCAGCACTAC	191
pCpGV-M_f-protein_r			TCCGCCACACTGTCCTTGAT	
pCpGV-M_vp39_f	*vp39*	late	TCCGGCAAGGACAATCGCTC	476
pCpGV-M_vp39_r			TGGCAGGTCAAACCCTCTG	
pCpGV-M_granulin_f	*gran*	very late	GGCCCGGCAAGAATGTAAGAATCA	422
pCpGV-M_granulin_r			GTAGGGCCACAGCACATCGTCAAA	

### Microarray design

**Fig. 1.**
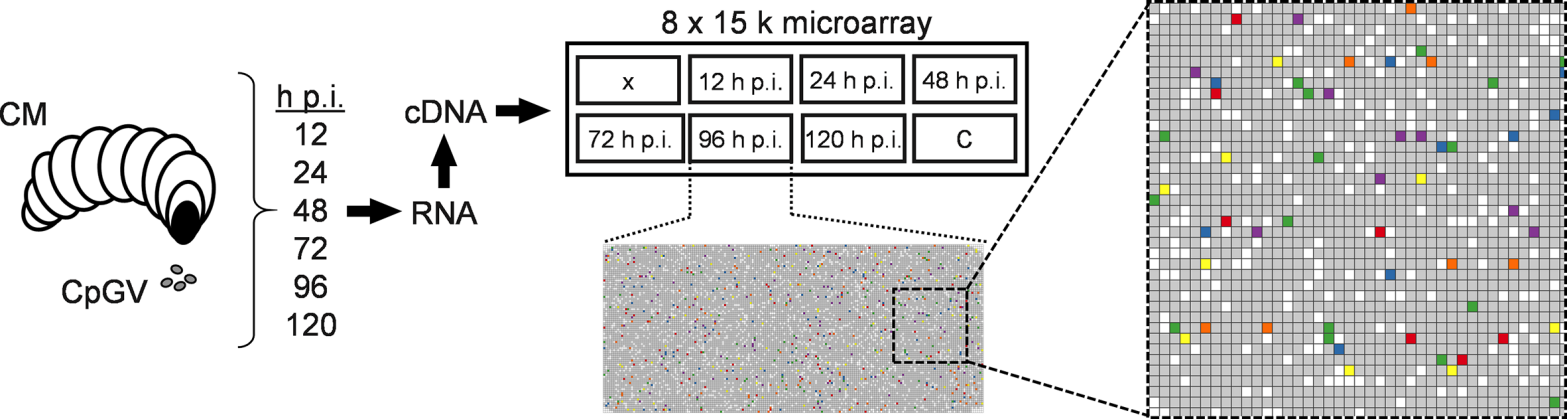
Workflow and design of a custom 8 x 15 k CpGV microarray. After peroral infection of CM larvae with CpGV occlusion bodies a subset of 30–50 larvae was removed at 12, 24, 48, 72, 96 and 120 h p.i. and subjected to midgut and fat body dissection. Uninfected larvae fed on virus-free control diet were collected at 120 h p.i. Dissection of midgut and fat body tissues was performed for ten larvae of each time point. Then, total RNA was isolated, reverse-transcribed to cDNA and analysed by a custom-designed 8 x 15 k microarray. Samples of six time points and the virus-free control (C) were hybridized to one the eight grids of the microarray, whereas one grid (X) remained unused. Each of the grids had 82 x 192 spots consisting of 100 technical replicates of randomly distributed oligonucleotides spots for 137 probed CpGV ORF, for actin (house-keeping gene), and for internal standard spike-ins and experimentally designed but unused probes. A part of the 96 h p.i. grid is enlarged for a more detailed view. As an example, the six marker genes used in the PCR analysis are highlighted: *ie-1* (red), *lef-8* (blue), *pe38* (green), *vp39* (purple), *f-protein* (orange) and *gran* (yellow). Grey: spots for probes of the remaining 131 ORF; white: spots for actin and other internal probes. After scanning, the data were processed for transcript quantification. A detailed description of the experiment is given in the Methods.

The transcriptome analysis of 137 (Table S1, available in the online verson of this article) of a total 142 annotated ORFs of CpGV-M [2, 37] was conducted by a 8×15 k microarray (Agilent) with custom-designed 60 bp oligonucleotides. Oligonucleotides were reverse complement (5′ to 3′ orientation) and located as close as possible to the 3′ end of potential transcripts of 137 ORFs (Table S1). Design of all specific 60mers with similar melting temperature was performed with eArray software v82.3.5.6 (Agilent). The house-keeping gene *actin* was chosen as standard for normalization. For five viral genes, i.e. *cp40*, *cp72*, *cp75*, *vlf-1* (*cp106)* and *me53* (*cp143*), appropriate 60mers could not be designed and did not result in analyzable transcription data. These genes were therefore not included in the study. All 60mers were checked for their specificity to CpGV-M and other entirely sequenced isolates of CpGV by multiple gene alignments using Geneious Prime (Biomatters, NZ) software. Spots for all oligonucleotides and internal standard spike-in (Agilent) were randomly distributed in 100-fold replicates, respectively (Fig. 1).

### Microarray cRNA preparation

For each infection experiment and its biological replicates, cDNA was prepared for microarray analysis. In total, 1.5 µl (50 ng µl^−1^) RNA were mixed with 2 µl spike-in RNA mix (Agilent), previously prepared according to the manufacturer’s instructions. Reverse transcription of the RNA/spike-in mix was performed with the Quick Amp Labelling Kit (Agilent) by combining 2 µl (5×) first strand buffer, 1 µl 0.01 M DTT, 0.5 µl 10 mM dNTP and 1.2 µl RNAse block mix to a total volume of 4.7 µl. After thorough mixing, the entire RNA/spike-in mix was added and cDNA was synthesized at 40 °C for 2 h. For labelling of transcripts, cDNA was converted into cRNA by the Quick Amp Labelling Kit (Agilent). To each cDNA probe, 0.75 µl H_2_O, 3.2 µl 5× transcription buffer, 0.6 µl (0.1 M) DTT, 1 µl (10 mM) dNTP mix, 0.21 µl T7-RNA-polymerase and 0.24 µl Cyanine 3-CTP were added and subsequently incubated at 40 °C for 2 h to generate Cy3 labelled cRNA. Labelled cRNA was purified from the reaction mixture by the RNA purification kit (Thermo Fisher) according the manufacturer’s protocol. The quality of the purified and labelled cRNA was measured by a NanoDrop 2000c (Peglab, Thermo Fisher). By measuring the Cy3 specific fluorescence, the ratio of pmol/Cy3 to µg RNA was measured and exceeded the required Cy3 activity of 6 pmol µg^−1^ RNA.

### Microarray analysis and data processing

For one microarray analysis, 600 ng of labelled cRNA were mixed with 5 µl 10× blocking agent, 1 µl 25× fragment buffer (Gene Expression Hybridization Kit, Agilent Technologies) and filled to a total volume of 25 µl. Incubation for 30 min at 60 °C resulted in fragmentation of cRNA. Samples were then immediately cooled on ice and mixed with 25 µl 2× GE hybridization buffer HI RPM. To avoid generation of bubbles, the mixture was centrifuged for 1 min at 13 000 r.c.f. and carefully loaded on the microarray for hybridization.

After pipetting the labelled and fragmented cRNA samples in the right spots onto the microarray, hybridization took place for 17 h at 65 °C in a hybridization oven. Non-hybridized cRNA fragments were removed from the microarray slide by three subsequent washing steps using wash buffer I, II and III (37 °C) for 1 min. After removing excessive liquid from the array slides, the microarrays were scanned with a resolution of 5 µm using an Agilent G2505C scanner (Agilent).

The obtained microarray images were processed by Feature Extraction Software v11.0 (Agilent) to transform signal intensities to numbers. The quality of each microarray was evaluated by the spike-in samples. Microarray raw data were processed with ArrayStar software version 5 (Lasergene, DNASTAR) for normalization with house-keeping gene *actin*. For each infection time point the three replicates were imported and processed together with quartile normalization. Genes were considered as transcribed when their *actin* normalized gene expression increased 1.5-fold higher than the background value of the control. Similar gene-expression patterns were identified with k-mean clustering in R (version 3.4.2).

For data analyses, ORFS of CpGV were allocated to temporal classes according to the presence of early (TATA box and CAGT motif), late and very late (A/T/G/TAAG motif) promoter motifs within 120 nt upstream of the ORF start codon in the genome of CpGV-M and to their predicted function according to gene homology [37–40].

## Results

### qPCR-based transcription analysis of selected genes in CpS and CpRR1

**Fig. 2.**
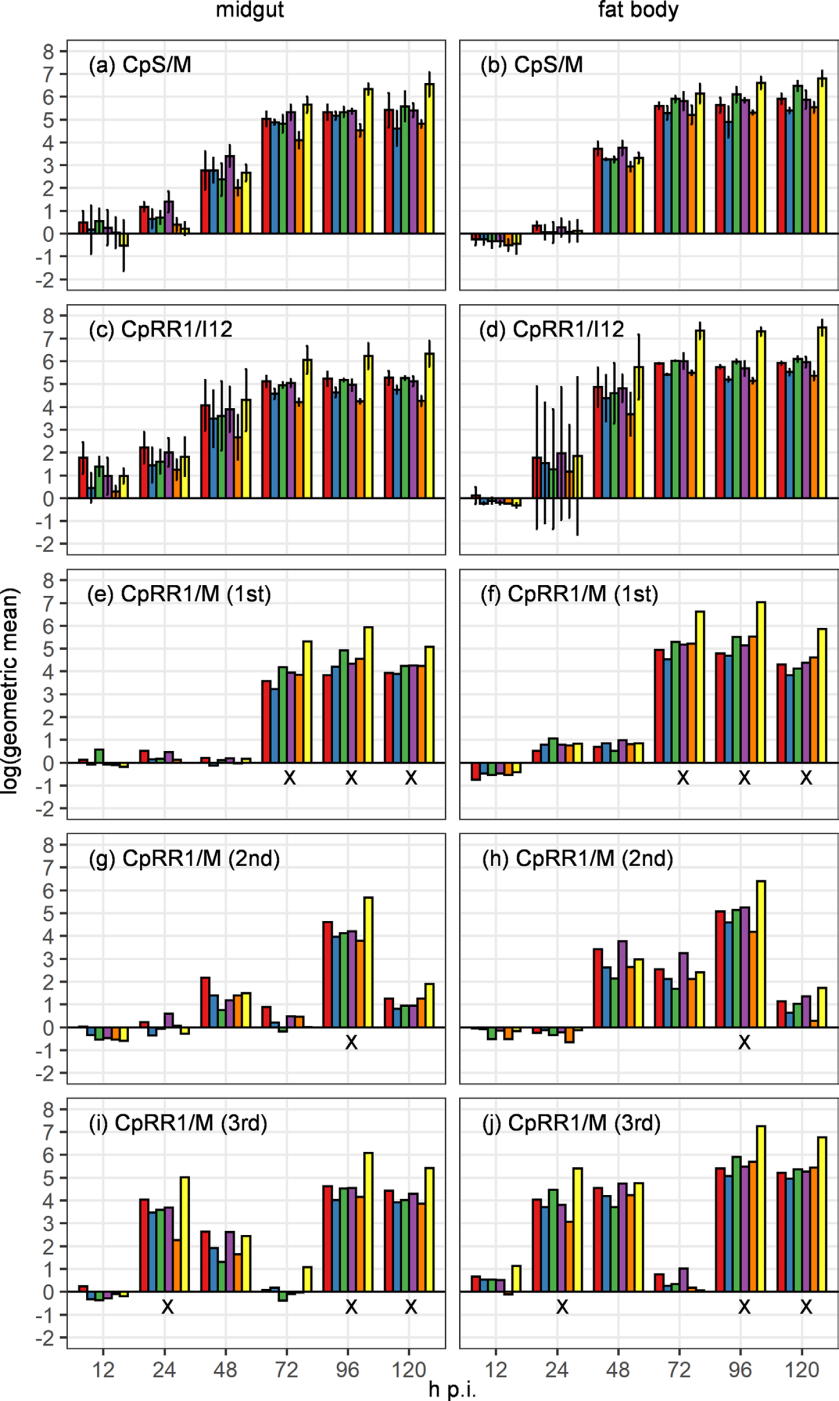
Transcription levels of *ie-1* (red), *lef-8* (blue), *pe38* (green), *vp39* (purple), *f-protein* (orange) and *gran* (yellow) in the midgut (left column) and fat body (right column) in permissive (a–d) and non-permissive (e–j) host/virus treatments determined by RT-qPCR as indicated in each panel. (a, b) Permissive CpS/CpGV-M treatment. (c, d) Permissive CpRR1/CpGV-I12 treatment. For the non-permissive treatment of CpRR1/CpGV-M the first (e, f), second (g, h) and third (i, j) replicates are shown separately. Vertical bars indicate +/- standard deviation. Time points of replicates considered as contaminated are marked with an "x" below the x-axis.

To analyse viral gene expression in midgut and fat body tissues of susceptible CpS and resistant CpRR1 larvae infected with either CpGV-M or CpGV-I12, the transcription of a set of six selected marker genes consisting of the immediate early (*ie-1* and *pe38*), early (*lef-8*), late (*f-protein* and *vp39*) and very late (*gran*) genes (Table 1) was investigated by RT-qPCR. Transcript abundance of the individual genes was determined at 12, 24, 48, 72, 96 and 120 h p.i. to examine temporal changes in gene expression. In CpGV-M-infected CpS larvae (CpS/CpGV-M), virus infection spreads from midgut to other tissues and thereby this experiment was considered the standard susceptible treatment. In this treatment, the primary infection was initiated in the midgut, which was measured by an onset in gene transcription of *ie-1*, *pe38*, *lef-8*, *f-protein*, *vp39* and *gran* from 12 to 120 h p.i. (Fig. 2a). Minimum gene expression was measured at 12 h p.i. and increased to 72 h p.i. At 72 h p.i. the gene expression was about 10^4^ to 10^6^-fold higher than the house-keeping gene *actin* for all measured genes and increased only slightly in the following time points at 96 and 120 h p.i. During this course of infection, the transcription level of *f-protein* was lowest when compared with the other genes, whereas transcription of *gran* increased continuously from 12 to 120 h p.i., and that of *lef-8* decreased from 96 to 120 h p.i. (Fig. 2a).

In fat body tissues of infected CpS larvae, transcription of CpGV-M genes was below the transcriptional level of *actin* (<1) at 12 h p.i. and exceeded the house-keeping gene threshold with values below 10-fold transcription at 24 h p.i. (Fig. 2b). Compared to the level of transcription of the midgut at 24 h p.i., the expression in the fat body was at a lower rate. At 48 h p.i., the gene expression of all six genes increased to levels between 10^2^ to 10^3^ and exceeded a 10^5^-fold higher expression than *actin* at 72, 96 and 120 h p.i. (Fig. 2b). Similar to the transcription in the midgut, the expression of *gran* peaked in comparison to *ie-1*, *pe38*, *lef-8*, *f-protein* and *vp39* at 96 and 120 h p.i. (Fig. 2b).

In a second experiment, the level of transcripts of the six selected marker genes were measured in midgut and fat body tissues of type I resistant CpRR1 larvae infected with CpGV-I12 (CpRR1/I12), a type I resistance-breaking isolate (Fig. 2c, d). Similar to CpS larvae infected with CpGV-M treatment, this can also be considered as a susceptible treatment, where an unimpeded viral infection was assumed. Viral transcripts for the marker genes were measured in the midgut (Fig. 2c) and fat body tissue (Fig. 2d) and the overall transcription pattern was similar to the CpS/CpGV-M treatment (Fig. 2a, b). The first transcripts were detected at 12 and 24 h p.i. in midgut (Fig. 2c) and fat body tissues (Fig. 2d), respectively, and increased to 72 h p.i. At 72, 96 and 120 h p.i., *ie-1*, *pe38*, *lef-8*, *f-protein* and *vp39* had a 10^4^- to 10^7^-fold higher level of transcription in midgut and fat body tissues, respectively, in comparison to *actin*. In both larval systems, CpS and CpRR1, the expression of *ie-1*, *pe38*, *lef-8*, *f-protein* and *vp39* was higher in the fat body than in midgut tissues at 72 to 120 h p.i. (Fig. 2a, b). For *gran*, the measured levels of transcription were similar in midgut and fat body tissues of CpGV-M-infected CpS larvae (Fig. 2a, b). In CpRR1 larvae infected with CpGV-I12, the amounts of *gran* transcripts exceeded a 10^7^-fold level in midgut and fat body tissues, respectively, at 72 to 120 h p.i., representing the highest values measured in all treatments and samples. These findings clearly indicated that CpGV-I12 successfully infected CpRR1 larvae.

In a next step, the larvae of CpRR1 were treated with CpGV-M to study viral gene expression of the six marker genes in resistant larvae treated with the resistance-prone CpGV-M. In this non-susceptible treatment CpRR1/CpGV-M, no steady increase in gene expression but high variability between time points was measured over time, contrasting the homogenous increase in the susceptible treatments CpS/CpGV-M and CpRR1/CpGV-I12 treatments. Because of these inconsistent transcriptional responses, all three replicates were analysed separately (Fig. 2e–j). Each replicate showed unique patterns of viral gene expression over time, which were similar within each replicate for the midgut and fat body tissues but highly different among the replicates. In the first replicate (Fig. 2e, f), the levels of gene expression from 12 to 48 h was close to zero and nearly always below 10-fold in both midgut and fat body tissues. Only at 72 to 120 h p.i. were higher levels of gene expression of all six marker genes detected and varied between 10^3^ to 10^7^ in both tissues. A different pattern was observed in the second and third replicate, where the abundance of transcripts peaked at 96 h p.i. (Fig. 2g, h) as well as at 24, 96 and 120 h p.i. (Fig. 2i, j), respectively. Two conclusions can be taken from these three replicates of non-susceptible treatment: (1) there are highly variable and inconsistent patterns of gene transcription and (2) the transcription level even at the time points of high transcription was 10 to 100-times lower than in the susceptible treatments noted for CpS/CpGV-M (Fig. 1a, b) and CpRR1/CpGV-I12 (Fig. 2c, d). As discussed below, the only biologically plausible explanation for this observation is that the pooled RNA samples of CpRR1/CpGV-M infections of single time points were contaminated with RNAs from individual CpRR1 larvae successfully infected with CpGV-M. Only when all CpRR1 larvae of a given time point were not productively infected with CpGV-M, then the transcription levels stayed low, as observed for the 48 h p.i. replicate no. 1, 72 and 120 h p.i. in replicate no. 2 and 72 h p.i. in replicate no. 3. Therefore, some of the samples were excluded from the following microarray analysis (Fig. 2).

### Microarray analysis of CpGV-M in susceptible CpS larvae

**Fig. 3.**
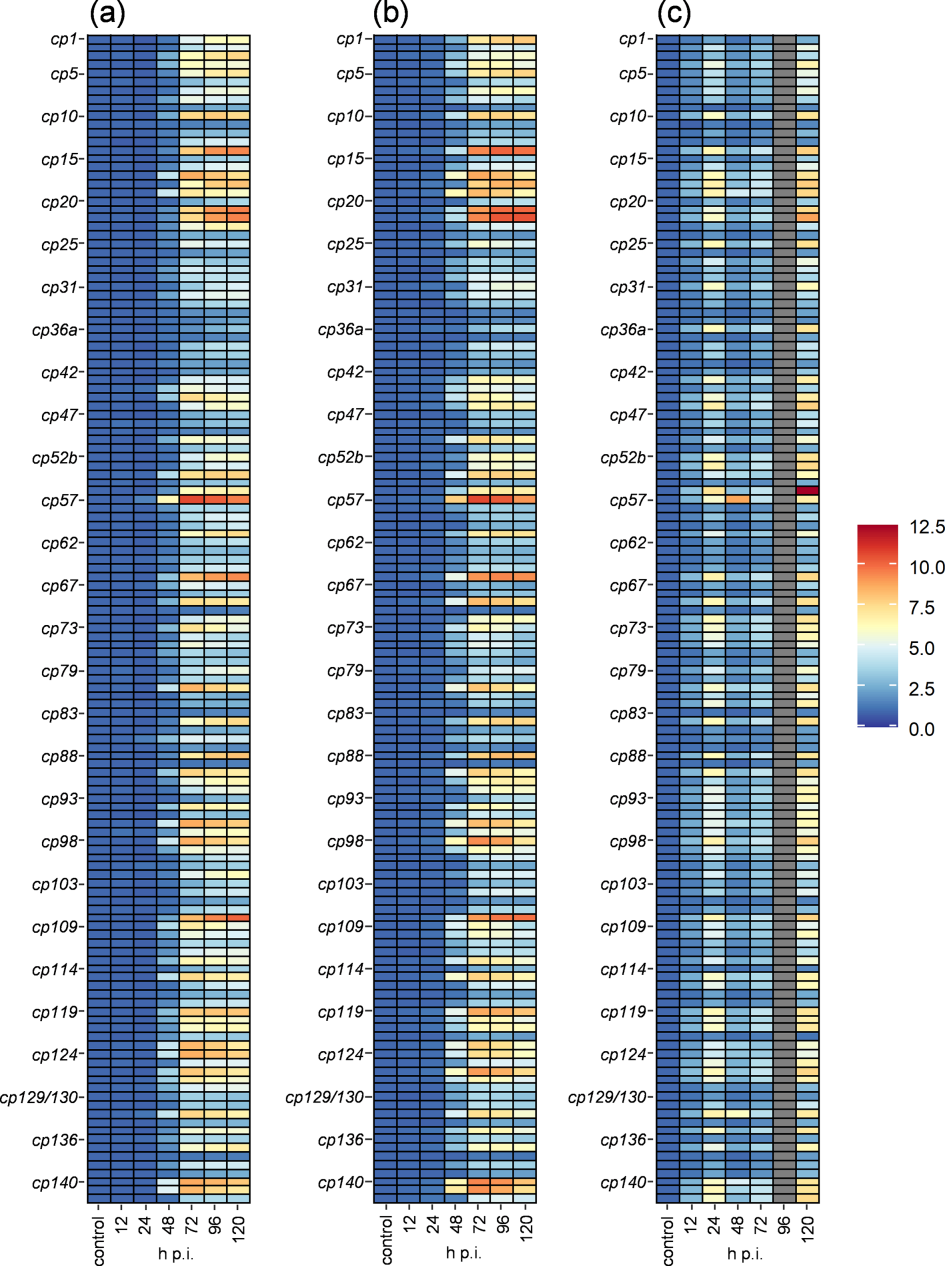
Heatmap of the temporal gene transcription within (a) midgut and (b) fat body tissue, both for CpS/CpGV-M as well as of (c) midgut of the CpRR1/CpGV-M treatment. Values based on geometric means of Table 2 but log_2_ transformed ranging from >0 (blue) to <12.5 (red) log_2_-fold change. No transcripts were measured in all replicates in the midgut of CpRR1 infected with CpGV-M at 96 h p.i. (grey). ORFs are to the left of the heatmaps and are numbered vertically from *cp1* to *cp142* (Table 2). ORF *cp40*, *cp72*, *cp75*, *cp106* and *cp143* were not included (see text for further information).

In a first step, the global transcription pattern of 137 genes of CpGV-M infecting CpS larvae was compared between midgut and fat body tissues (Table 2, Fig. 3a, b). For the entire microarray analysis, RNA preparations from midgut and fat body tissues of non-infected CpS larvae, collected at 120 h p.i., served as the internal reference to which the levels of CpGV-M transcription were normalized. In the course of infection, the levels of gene transcripts increased from 12 to 120 h p.i. (Fig. 3). At 12 and 24 h p.i., the expression was low and did not exceed the 2.6-fold increase of *p6.9* (*cp86*) and 1.4-fold increase of several genes in the midgut and fat body tissues, respectively (Table 2). In both tissues, none of the 137 ORFs showed evidence of transcription at 12 or 24 h p.i. An onset in gene expression was observed at 48 h p.i., when *pp31* (*cp57*) had a 203 and 2970-fold higher transcript abundance in the midgut and fat body, respectively. The second highest expressed gene was *p6.9* (62.2-fold) followed by 21 ORFs with expression levels between 10.5- to 48.1-fold. The levels of transcripts of the remaining 114 ORFs were below the 10-fold expression of the untreated control. In the fat body, *egt* (*cp141*) (483-fold) followed *pp31* as the second highest expressed gene and 18 ORFs were measured with transcripts being 101- to 371-fold higher expressed. The remaining 50 and 67 ORFs measured transcript levels from 10- to 100-fold and below 10-fold, respectively. Neither in the midgut nor in the fat body tissue, did ORFs exhibit maxima of gene transcription at 12, 24 or 48 h p.i. At 72 h p.i., 49 and 50 ORFs had their temporal maximum in the midgut and fat body, respectively, whereas 48 of these ORFs shared their maximum in both tissues. Only *cp79*, *dnapol* (*cp111*) and *cp119* had their maximum abundance of transcripts in either the midgut or the fat body at 72 h p.i. The gene with the highest level of transcription in comparison with all other time points in the entire microarray analysis was *pp31* (*cp57*) with a 14 400 and 88 300-fold increase in the midgut and fat body at 72 h p.i., respectively (Table 2, Fig. 3). The other very highly expressed gene in the midgut was *p6.9* (*cp86*), encoding for the DNA-binding protein. In the fat body the second highest expressed gene was *ubiquitin* (*cp54*) with a 13 000-fold abundance (Table 2). In the last two measured time points at 96 and 120 h p.i., *pp31* was the highest transcribed gene, although its level of transcription decreased steadily. In the following course of infection, 18 and 70 ORFs peaked in their transcription at 96 and 120 h p.i., respectively, in the midgut, whereas in the fat body 72 and 15 ORFs reached their maximum at 96 and 120 h p.i., respectively. Most of those 70 ORFs which had their maximum at 120 h p.i. in the midgut have their maximum at 72 h p.i. in the midgut (Table 2) indicating a shift in the maximal transcript rate in the midgut and fat body tissue from 120 h p.i. to 96 h p.i.

**Table 2.** Relative transcription values of structural (struc), regulatory (reg) and auxiliary (aux) CpGV-M genes in CpS/CpGV-M (midgut and fat body) CpRR1/CpGV-M (midgut) treatments at 12, 24, 48, 72, 96 and 120 h p.i. Microarray transcription values of each replicate were normalized to the host’s *actin* transcription levels. The replicate values were summarized by creating the geometric mean and then normalized by dividing by the uninfected control. Thereby, the values reflected the increase in viral gene expression in relation to the control. Maxima were marked in bold font and highlighted in grey. Core genes are underlined. Promoters (Prom.): e=early (TATA box and CAGT motif) and l=late (A/T/G/TAAG motif) were identified within 120 nt upstream of the corresponding ORF start codon [2, 38, 39]. In the CpRR1/CpGV-M (fat body) treatment viral transcription was not abundant and is therefore not shown.

ORF	Gene	Prom.	Func.	Cluster		CpS+CpGV M (midgut)						CpS+CpGV M (fat body)						CpRR1+CpGV M (midgut)				
				Midgut	Fat body	h p.i.						h p.i.						h p.i.				
						12	24	48	72	96	120	12	24	48	72	96	120	12	24	48	72	120
*cp1*	*gran*	l	struc	D	D	1.0	0.7	1.6	381.6	1951.0	**2095.7**	1.0	1.1	11.8	5048.0	13387.1	18322.8	1.5	3.7	1.2	3.1	**4.7**
*cp2*		e, l		D	D	1.0	0.9	1.3	25.5	69.2	**92.3**	1.1	1.3	2.4	83.3	**147.6**	77.2	1.7	5.8	1.7	2.9	**9.4**
*cp3*	*pk1*	e	struc	B	D	1.2	0.9	7.3	716.5	2291.4	**4033.9**	1.4	1.3	38.3	3658.8	9795.9	12295.0	1.6	4.1	1.9	3.5	**7.9**
*cp4*		e, l		A	A	1.0	0.9	7.7	**310.9**	227.6	184.8	1.0	1.1	70.1	**1254.6**	939.9	322.8	1.7	5.2	1.9	2.8	**8.0**
*cp5*				D	D	1.0	0.8	2.2	256.5	898.2	**1215.0**	1.0	1.2	24.2	2262.0	4711.8	**6373.4**	1.6	5.4	1.8	2.9	**10.4**
*cp6*		l		D	C	1.1	0.9	1.3	12.0	35.1	**41.2**	1.1	1.3	1.7	62.8	**132.1**	118.8	1.6	5.7	1.7	2.8	**10.1**
*cp7*	*ie-1*	e	reg	D	D	0.9	0.9	1.4	59.6	146.1	**163.6**	1.0	1.2	6.9	344.9	**734.4**	602.6	1.6	6.4	1.8	3.0	**11.9**
*cp8*	*ac146*	e	struc	A	B	1.1	0.9	5.1	**235.8**	135.8	66.9	1.0	1.1	36.8	**652.5**	374.4	96.0	1.5	4.6	1.6	2.9	**5.5**
*cp9*	*ac145*	e, l	struc	C	C	1.0	1.1	1.9	9.6	**33.9**	33.0	1.2	1.0	1.1	14.6	**40.5**	34.0	1.8	1.2	1.1	2.4	**5.1**
*cp10*	*chitinase*	e	aux	B	A	1.1	0.9	4.0	432.8	**567.4**	396.1	1.1	1.3	38.1	1852.2	**1915.1**	1008.8	1.7	4.6	1.8	2.6	**6.6**
*cp11*	*cathepsin*	l	aux	C	D	1.2	1.2	2.7	8.7	18.5	**21.3**	0.7	0.9	4.4	73.1	**145.7**	110.3	0.8	5.7	1.5	2.7	**10.8**
*cp12*		l		B	D	1.0	0.9	2.7	**57.1**	56.9	54.9	1.0	1.1	9.2	**232.5**	202.0	109.8	1.5	9.6	1.8	4.2	**18.4**
*cp13*	*gp37*	l	aux	D	D	0.7	0.8	1.7	201.7	**567.5**	520.7	1.0	0.8	5.2	748.5	**1196.8**	927.6	1.4	2.3	1.3	2.5	**2.8**
*cp14*	*odv-e18*	l	struc	D	D	1.0	0.9	1.9	500.1	1703.2	**2031.0**	1.1	1.4	24.3	4589.7	**9472.3**	7057.0	1.7	4.6	1.7	2.6	**6.7**
*cp15*	*p49*	e, l	struc	D	D	1.0	0.8	1.3	34.6	91.6	**111.0**	0.8	0.8	2.8	166.6	**307.8**	262.9	1.4	5.8	1.5	2.9	**11.7**
*cp16*		e		D	D	1.0	0.8	2.4	64.9	148.4	**193.7**	1.0	1.1	11.9	316.7	**587.7**	548.5	1.7	5.4	1.8	2.9	**8.6**
*cp17*	*iap-3*	e	reg	A	A	1.0	1.0	20.3	**1162.5**	669.8	368.3	1.1	1.3	242.4	**5611.6**	3378.3	675.1	1.7	4.9	1.9	2.7	**6.5**
*cp18*	*odv-e56*	e, l	struc	D	C	1.0	0.9	1.3	102.4	446.6	**560.0**	1.1	1.4	2.2	895.8	1998.4	**1909.3**	1.8	4.8	1.8	2.6	**6.7**
*cp19*	*orf15R*	e		A	A	1.0	0.9	35.1	**492.4**	277.5	164.4	1.1	1.3	308.7	**2528.6**	1512.2	327.5	1.8	5.3	3.1	2.8	**7.3**
*cp20*	*orf16L*	l		C	C	0.9	0.9	1.1	11.5	48.3	**82.5**	0.9	0.9	1.3	92.1	**237.0**	217.1	1.5	7.8	1.7	3.7	**14.5**
*cp21*	*orf17L*			D	D	1.0	0.9	2.4	854.4	3891.2	**6136.7**	1.1	1.3	29.4	8976.9	25168.5	27879.0	1.7	4.9	1.7	2.7	**10.0**
*cp22*	*orf17R*	l	struc	D	D	1.0	0.9	2.2	666.0	2803.6	**4605.1**	1.1	1.3	23.4	7407.5	21071.6	23034.7	1.8	5.2	1.7	2.7	**13.1**
*cp23*	*pe/pp34*	e, l	struc	D	D	0.9	0.8	3.5	1060.7	4536.6	**6425.9**	0.9	1.0	14.7	4983.1	14523.7	12766.4	1.6	5.3	1.9	3.4	**21.0**
*cp24*	*pe38*	e	reg	A	A	1.0	0.9	6.5	**51.5**	35.7	35.3	1.0	1.0	11.5	**84.8**	46.9	22.5	1.5	7.5	1.8	3.3	**9.3**
*cp25*		e		B	A	1.0	0.9	1.7	**46.3**	30.3	29.1	1.1	1.4	11.2	**193.3**	113.0	53.7	1.7	4.9	1.7	2.5	**6.5**
*cp26*		l		D	C	0.9	1.0	1.6	18.5	59.7	**71.4**	1.0	0.8	1.5	66.4	161.1	**175.8**	1.5	5.5	1.2	3.6	**19.2**
*cp27*		e		B	B	1.0	0.9	2.0	**25.1**	16.4	17.1	1.0	1.2	8.1	**66.9**	43.1	18.5	1.7	6.2	1.8	2.7	**9.1**
*cp28/29*	*1*	e		A	A	0.9	1.1	3.7	**60.2**	40.9	31.4	0.9	0.8	14.0	**138.8**	106.0	55.0	1.4	3.7	1.6	2.7	**7.1**
*cp30*		e		B	A	0.8	0.8	4.8	146.5	**168.2**	162.4	0.8	0.9	39.1	930.5	**1081.4**	626.7	1.5	8.0	2.1	3.4	**11.8**
*cp31*	*f-protein*	e	struc	C	C	1.0	0.9	1.3	8.9	24.5	**36.5**	1.1	1.4	1.5	70.8	**153.1**	148.6	1.7	5.6	1.7	2.8	**6.2**
*cp32*		l		B	A	1.0	0.9	6.3	201.9	274.0	**292.5**	1.1	1.1	30.4	927.1	**1070.5**	675.4	1.5	6.0	2.0	3.2	**12.9**
*cp33*		l		D	D	1.0	1.0	1.9	44.2	89.2	**123.1**	0.9	0.8	3.7	154.2	**268.1**	199.8	1.4	4.0	1.3	3.1	**4.4**
*cp34*		e		B	C	1.0	1.0	1.6	4.7	**5.8**	5.7	1.1	1.1	1.0	5.5	**8.4**	5.1	1.5	2.4	1.2	2.7	**8.7**
*cp35*	*pif-3*	e, l	struc	C	C	0.9	0.9	1.5	2.5	6.6	**10.3**	0.8	0.8	1.3	4.9	**12.0**	10.4	1.4	**4.2**	1.5	3.1	3.0
*cp36a*		e		C	C	1.0	0.9	1.2	2.3	4.9	**8.2**	1.1	1.3	1.4	8.5	21.7	**25.1**	1.7	4.8	1.7	2.6	**6.5**
*cp36b*		e		C	C	0.9	1.0	1.7	2.1	3.7	**5.4**	0.9	0.8	0.9	2.4	**4.9**	4.1	1.5	2.2	1.2	2.6	**7.9**
*cp37*	*odv-e66*	l	struc	D	D	1.0	0.8	1.2	23.6	**65.8**	65.0	1.1	1.2	2.2	162.6	**282.0**	204.3	1.6	6.3	1.8	3.3	**11.2**
*cp39*		l		D	D	1.1	0.8	1.5	21.5	54.6	**60.4**	0.9	1.0	2.3	83.2	**180.0**	128.2	1.7	10.6	1.9	3.5	**14.2**
*cp41*	*lef-2*	e	reg	B	A	0.8	1.0	3.7	**35.9**	34.9	31.0	1.1	0.8	6.0	**77.3**	67.7	28.7	1.7	2.5	1.0	1.8	**10.6**
*cp42*	*orf35a*	e		D	D	1.0	0.7	1.0	13.9	26.4	**29.6**	1.3	1.0	2.6	67.1	**94.3**	60.6	1.6	7.7	1.8	3.6	**26.4**
*cp43*		e, l		B	D	0.9	0.8	1.5	74.9	**92.0**	72.3	1.1	1.3	11.4	502.7	**508.0**	272.5	1.7	5.1	1.8	2.7	**7.1**
*cp44*	*orf36L*	e, l		A	A	1.1	1.3	44.7	**1601.4**	857.6	423.4	1.1	1.2	275.7	**7454.6**	4329.0	823.4	1.7	8.1	4.4	3.7	**16.6**
*cp45*		e		A	A	1.1	0.9	10.5	**641.2**	316.7	132.4	1.1	1.2	100.9	**2252.6**	1332.2	233.2	1.8	4.8	1.8	2.7	**7.0**
*cp46*	*mp-nase*		struc	D	D	1.0	0.9	1.3	40.8	82.1	**87.0**	1.1	1.4	7.2	256.8	**400.5**	331.1	1.8	5.1	1.8	2.7	**6.7**
*cp47*	*p13*	l	struc	D	C	0.8	0.8	1.2	9.9	21.0	**25.0**	0.9	1.0	1.5	45.7	**79.2**	54.6	1.5	6.7	1.7	3.3	**12.0**
*cp48*	*pif-1*		struc	C	C	1.3	1.1	1.5	5.6	10.6	**13.1**	1.1	1.3	1.3	19.1	**34.6**	31.4	1.6	4.8	1.8	3.1	**8.5**
*cp49*		l		C	C	1.1	0.9	1.4	3.1	4.7	**5.7**	1.1	1.2	1.4	7.7	**12.0**	9.1	1.6	10.3	1.7	4.3	**16.2**
*cp50/51*		l		B	A	0.9	0.9	6.7	**387.2**	333.2	210.5	1.0	1.2	77.9	**2063.2**	1399.0	586.2	1.6	5.4	2.0	2.7	**8.1**
*cp52a*	*ac106/107*	l		D	D	1.0	0.9	1.7	56.4	158.9	**203.5**	1.1	1.1	2.5	203.2	**428.3**	388.7	1.6	10.4	1.6	4.0	**14.5**
*cp52b*		e		D	D	1.0	0.9	1.2	61.7	186.0	**231.3**	1.0	1.2	3.3	467.4	**923.3**	680.3	1.7	4.8	1.7	2.6	**7.0**
*cp53*	*ac110*	e		D	C	1.0	0.9	1.3	14.8	**38.9**	32.1	1.1	1.4	1.6	103.1	**167.6**	101.6	1.7	5.1	1.8	2.6	**6.6**
*cp54*	*ubiquitin*		aux	A	A	1.1	1.1	35.9	2163.0	**3326.0**	3317.3	1.0	1.1	275.5	13032.6	16028.4	10058.1	1.6	5.0	3.3	3.0	**9.8**
*cp55*	*odv-ec43*	e, l	struc	B	D	1.2	0.8	2.8	83.0	129.0	**143.6**	1.2	1.1	6.5	200.2	**314.8**	233.1	1.5	5.6	2.6	3.8	**15.4**
*cp56*	*ac108*	l	struc	B	D	0.9	0.9	2.5	206.4	311.4	**355.5**	0.9	1.1	27.0	1238.9	**1912.7**	1349.2	1.8	5.9	1.8	2.5	**62.2**
*cp57*	*pp31/39K*	e	reg	A	A	1.0	1.7	203.3	14431.6	9202.9	5013.8	1.2	1.3	2972.7	88318.5	58773.9	14348.9	1.7	5.0	**12.9**	2.9	6.7
*cp58*	*lef-11*		reg	B	A	1.2	0.9	6.5	**244.4**	222.3	198.3	1.1	1.2	30.1	**834.0**	792.7	395.5	1.5	10.9	1.9	4.9	**16.1**
*cp59*	*sod*	l	aux	D	D	1.0	1.0	1.5	27.1	72.9	**77.4**	0.9	0.9	2.4	86.6	**158.7**	130.4	1.5	4.0	1.5	3.3	**5.8**
*cp60*	*p74*	l	struc	C	C	1.1	0.9	1.6	23.1	79.0	**146.2**	0.9	1.2	1.2	49.8	123.5	**139.2**	1.4	4.9	1.3	3.5	**6.0**
*cp61*		l		D	D	1.0	0.9	1.7	136.9	374.8	**567.5**	1.0	1.2	13.7	1100.6	**2098.9**	1875.6	1.6	5.2	1.7	2.6	**6.7**
*cp62*		e, l		D	D	1.0	0.8	2.6	131.8	398.5	**633.5**	0.9	0.9	7.8	585.7	**1161.0**	1055.7	1.5	7.1	1.8	3.9	**14.0**
*cp63*	*bro*	e	reg	B	D	0.9	0.8	1.6	20.1	**28.8**	19.6	1.1	1.0	3.7	75.9	**85.7**	44.6	1.5	4.7	1.5	3.1	**9.2**
*cp64*		e, l		B	D	1.0	1.1	4.7	130.0	170.8	**189.0**	1.0	1.0	13.3	399.5	**427.6**	239.8	1.5	6.7	1.7	3.9	**10.9**
*cp65*		l		D	D	1.1	0.9	1.7	61.8	111.3	**129.5**	1.2	1.3	4.9	239.8	**268.3**	151.0	1.6	4.9	1.6	3.3	**11.7**
*cp66*	*ptp-2*		struc	B	A	1.0	0.9	7.2	736.1	1393.8	**1851.1**	1.1	1.4	92.6	4938.2	**6811.1**	5671.5	1.7	5.0	1.8	2.6	**6.6**
*cp67*		l		A	B	1.1	1.2	8.9	**276.6**	189.7	121.4	0.8	1.0	17.4	**249.6**	135.4	35.4	1.5	3.5	1.8	3.3	**9.2**
*cp68*	*p47/pif-5*	e	reg	D	D	0.9	0.9	1.9	43.5	110.5	**148.5**	0.8	1.0	3.7	164.0	**299.4**	247.9	1.4	6.7	1.6	3.7	**10.3**
*cp69*		l		B	A	1.0	0.9	4.6	**323.6**	265.9	210.7	1.1	1.4	67.1	**1716.2**	1266.1	699.4	1.7	4.9	1.7	2.7	**6.5**
*cp70*		l		C	C	1.0	0.9	1.4	2.0	2.7	**3.6**	0.8	1.0	0.9	2.8	**4.4**	2.6	1.4	10.5	1.5	4.5	**17.1**
*cp71*	*p24capsid*	l	struc	D	D	0.9	0.8	1.1	99.3	310.6	**403.2**	0.9	1.0	3.3	683.5	**1334.3**	1105.1	1.7	5.2	1.8	2.7	**7.1**
*cp73*	*38.7K*	e		A	B	1.0	0.9	5.9	**372.1**	157.6	59.7	1.0	1.1	78.7	**1262.9**	485.7	63.6	1.7	5.0	1.7	2.8	**6.3**
*cp74*	*lef-1*	e	reg	B	B	1.1	0.9	1.5	**39.9**	22.1	13.5	1.1	1.2	9.4	**122.8**	69.6	16.4	1.7	4.9	1.7	2.7	**7.0**
*cp76*	*fgf-1*	e	aux	A	B	1.1	0.9	4.2	**146.4**	89.6	43.8	0.9	1.2	33.0	**734.7**	447.3	67.5	1.6	5.2	1.8	2.8	**6.6**
*cp77*		e		A	B	0.9	1.0	13.4	**258.9**	118.3	60.2	0.9	0.9	73.9	**968.4**	578.5	81.5	1.5	9.7	2.4	4.4	**16.0**
*cp78*		e		B	B	1.1	0.9	3.4	47.4	**48.3**	37.4	1.2	1.1	8.6	**82.6**	72.8	23.6	1.6	6.0	1.8	3.4	**12.2**
*cp79*		l		D	C	1.1	0.9	1.3	18.3	60.1	**76.4**	1.2	1.3	1.8	89.1	205.2	**208.1**	1.7	5.0	1.7	2.8	**7.7**
*cp80*	*lef-6*	e	reg	B	B	1.1	0.9	3.0	**61.9**	46.0	26.7	1.0	1.1	15.1	**223.9**	168.1	39.6	1.5	4.9	1.7	2.9	**11.8**
*cp81*	*dbp*	e	reg	A	A	1.1	0.9	21.4	**1047.2**	538.4	252.0	1.1	1.3	211.1	**4823.8**	2537.6	543.0	1.7	4.8	2.3	2.6	**7.6**
*cp82a*	*82a*	e		B	B	0.9	0.8	1.2	**15.6**	9.0	4.9	1.0	1.1	4.6	**80.5**	55.2	13.9	1.6	4.6	1.6	2.8	**8.0**
*cp82b*	*82b*	e		B	C	1.0	0.9	1.3	4.6	4.4	**4.8**	1.1	1.3	1.5	17.4	**17.6**	8.2	1.7	5.5	1.7	2.8	**8.4**
*cp83*	*p45(p48)*	e	struc	C	C	0.9	1.0	1.7	5.1	12.8	**17.5**	1.0	0.9	1.0	14.5	**28.8**	24.9	1.4	3.1	1.0	3.8	**10.6**
*cp84*	*p12*	l	struc	D	D	1.0	0.9	1.6	124.2	381.9	**584.6**	1.1	1.3	11.4	945.7	**2137.8**	1923.3	1.8	5.1	1.8	2.8	**7.1**
*cp85*	*bv/odv-c42 (p40)*	e, l	struc	B	D	0.9	0.9	3.1	44.1	56.3	**66.3**	0.9	0.9	5.6	131.8	**168.9**	127.8	1.5	12.9	1.6	4.7	**18.3**
*cp86*	*p6.9*	l	struc	A	A	1.2	2.6	62.2	**3009.1**	1907.7	1230.6	1.0	1.3	204.9	**6760.2**	5092.2	1455.8	1.5	9.6	4.4	4.4	**11.7**
*cp87*	*lef-5*		reg	A	B	0.9	1.0	2.5	**17.6**	11.6	7.1	1.1	0.9	3.1	**32.3**	16.4	3.8	1.5	3.0	1.2	3.2	**7.8**
*cp88*	*38* k		struc	D	D	1.0	0.9	1.9	215.4	530.8	**665.5**	1.1	1.4	17.0	1529.1	**2671.8**	2117.4	1.7	5.0	1.7	2.8	**6.5**
*cp89*	*pif-4*	l	struc	C	C	0.9	0.9	1.6	2.2	3.5	**4.0**	0.9	1.0	0.9	2.8	**4.9**	2.7	1.5	2.1	1.0	3.1	**13.9**
*cp90*	*helicase*	l	reg	A	A	1.0	0.9	8.9	**543.0**	353.3	248.8	1.1	1.3	113.8	**2169.2**	1456.5	516.4	1.7	5.1	1.8	2.6	**6.6**
*cp91*	*odv-e25*	e, l	struc	D	D	1.0	0.8	3.7	654.6	1956.7	**2731.7**	0.9	1.0	30.6	4369.9	**9598.0**	9038.5	1.6	6.7	2.1	3.3	**18.1**
*cp92*	*p18*	l	struc	D	C	1.0	0.9	1.3	23.3	70.6	**90.8**	1.1	1.2	1.8	184.0	**395.7**	300.7	1.7	5.2	1.7	2.7	**8.0**
*cp93*	*p33*	e, l	struc	C	C	0.9	0.8	1.1	3.0	8.8	**13.9**	1.1	1.3	1.4	8.8	35.2	**43.6**	1.7	5.3	1.7	2.7	**8.7**
*cp94*	*iap*	l	reg	A	A	1.0	0.9	10.8	**461.5**	310.5	216.2	0.9	1.1	94.3	**1840.8**	1187.5	377.5	1.6	4.8	1.7	2.4	**6.2**
*cp95*	*lef-4*	l	reg	B	D	0.9	0.8	1.2	**21.3**	13.9	12.1	1.0	1.2	4.7	**92.9**	70.3	32.5	1.7	5.4	1.8	2.7	**8.0**
*cp96*	*vp39*	l	struc	A	A	1.1	1.0	26.5	**1238.7**	835.9	594.7	1.1	1.3	225.5	**5677.2**	4491.3	1072.2	1.7	4.8	2.7	2.6	**7.3**
*cp97*	*odv-ec27*	e, l	struc	B	D	1.3	1.1	3.4	178.3	303.6	**349.6**	1.0	1.1	20.1	854.1	**1305.3**	816.7	1.8	6.1	1.8	2.9	**7.7**
*cp98*	*ptp*	e	struc	A	A	1.0	0.9	25.6	**1047.1**	564.7	283.0	1.1	1.4	231.4	**4977.0**	3254.5	536.5	1.7	4.8	1.9	2.6	**6.6**
*cp99*		e		B	A	1.0	0.9	4.6	**186.4**	148.6	114.1	1.0	1.2	40.6	**944.0**	698.8	274.4	1.7	5.3	1.9	2.9	**8.2**
*cp100*		l		C	C	1.0	0.8	1.2	11.5	47.8	**67.9**	1.0	1.2	1.5	87.7	**206.5**	196.8	1.6	4.8	1.7	2.6	**8.4**
*cp101*	*vp91*	e, l	struc	D	C	1.1	1.3	1.5	9.5	17.5	**19.7**	0.9	1.0	1.0	20.1	**33.5**	22.9	1.4	**4.5**	1.4	2.8	4.5
*cp102*	*tlp20*	l		D	D	1.2	1.0	1.6	56.0	111.2	**162.4**	1.2	1.2	5.0	194.1	**332.4**	317.0	1.6	5.7	1.7	2.9	**6.7**
*cp103*	*ac81*	l		C	C	1.0	0.9	1.3	6.8	19.9	**30.5**	1.0	1.0	1.0	22.5	**52.2**	49.2	1.5	4.5	1.5	3.5	**8.1**
*cp104*	*gp41*	l	struc	D	D	1.0	0.8	1.2	41.2	139.1	**199.5**	1.0	1.1	2.4	279.8	**576.3**	542.8	1.7	5.3	1.8	3.3	**12.1**
*cp105*	*ac78*	l		C	C	0.8	1.0	1.5	10.5	32.0	**40.1**	1.1	0.8	1.4	26.6	**52.5**	47.0	1.5	2.5	1.3	2.3	**2.7**
*cp107*		e, l		D	D	1.1	0.9	1.6	77.4	244.4	**333.2**	1.0	1.0	3.0	338.5	**741.4**	657.5	1.6	9.4	1.8	4.1	**16.3**
*cp108*	*ac75*	l	struc	D	D	1.0	0.9	3.9	1480.1	4290.0	**8503.2**	1.0	1.3	52.7	10271.9	22661.6	25271.3	1.7	5.0	1.8	2.6	**7.2**
*cp109*				A	B	1.0	0.9	23.1	**566.6**	261.8	108.6	1.0	1.2	187.5	**1169.5**	382.1	46.9	1.6	4.8	2.1	2.8	**5.9**
*cp110*		e		B	A	1.0	0.9	4.1	**117.3**	75.5	60.6	1.0	1.2	40.0	**414.3**	306.4	129.9	1.7	5.6	1.8	2.7	**8.9**
*cp111*	*dnapol*		reg	B	A	1.0	0.8	2.5	**42.1**	36.0	25.1	1.0	1.0	14.7	162.6	**163.4**	66.7	1.6	6.5	1.9	3.7	**15.4**
*cp112*	*desmoplakin*	e	struc	B	D	1.0	0.9	3.6	180.7	319.5	**360.6**	0.7	0.9	14.6	512.2	**712.7**	613.4	1.5	5.2	1.6	3.0	**9.2**
*cp113*	*lef-3*	e	reg	A	A	1.0	0.9	8.6	**376.8**	252.9	155.1	1.1	1.2	101.9	**2184.9**	1337.3	406.2	1.7	5.6	2.0	2.9	**8.2**
*cp114*	*pif-6*	e	struc	D	D	0.8	1.0	1.5	34.6	128.5	**222.0**	1.1	0.8	2.4	119.3	293.0	**321.0**	1.6	2.4	1.3	2.4	**2.9**
*cp115*		e		A	A	1.0	0.9	21.0	**521.1**	273.5	248.5	1.0	1.3	273.1	**2458.2**	1661.2	618.5	1.7	5.3	2.3	2.6	**6.6**
*cp116*	*iap-5*	e	aux	C	C	1.0	0.9	1.3	12.3	41.3	**58.1**	1.0	1.2	1.8	91.0	188.8	**197.7**	1.7	5.4	1.8	2.8	**8.2**
*cp117*	*lef-9*	l	reg	D	D	1.2	1.1	2.0	20.7	31.8	**34.6**	0.9	1.1	2.2	39.4	**55.5**	45.0	1.5	8.6	1.6	3.5	**12.3**
*cp118*	*fp25k*	l	struc	D	D	1.0	0.9	3.4	146.6	339.9	**417.3**	0.9	1.0	9.1	463.8	**809.8**	649.7	1.7	7.6	1.8	3.8	**13.9**
*cp119*		e		B	A	1.0	0.9	6.4	553.4	**633.6**	619.4	1.1	1.4	77.6	**2908.8**	2715.0	1501.2	1.7	5.1	1.8	2.7	**6.5**
*cp120*	*DNA ligase*		reg	B	A	1.0	0.9	4.7	**199.5**	146.0	103.4	1.1	1.3	38.8	**811.3**	568.5	182.1	1.7	5.4	1.9	2.8	**8.2**
*cp121*				B	D	1.0	0.9	2.7	131.1	**195.1**	181.8	1.1	1.3	19.8	627.7	**814.2**	562.5	1.8	5.5	1.9	2.9	**7.9**
*cp122*		e		A	B	0.8	1.0	7.4	**193.0**	139.1	97.0	0.8	1.0	10.4	**252.3**	173.7	51.0	1.6	**3.8**	1.3	2.6	1.7
*cp123*	*fgf*		aux	A	A	1.1	1.1	28.6	**1582.6**	983.9	369.0	1.1	1.3	277.2	**6552.6**	3734.7	586.4	1.7	5.7	3.0	2.6	**8.4**
*cp124*		e		A	A	1.1	1.0	23.9	**1432.5**	997.1	623.8	1.1	1.2	164.4	**6921.5**	5454.5	1116.2	1.6	4.2	2.5	2.7	**6.0**
*cp125*	*alk-exo*	e	aux	B	D	1.1	1.0	1.5	31.2	38.4	**38.5**	1.0	1.2	4.0	111.8	**138.3**	97.2	1.8	5.0	1.8	2.9	**7.4**
*cp126*	*helicase-2*	e	reg	A	A	1.0	0.9	13.2	**923.8**	588.8	346.4	1.1	1.3	209.7	**5166.0**	3255.4	1044.0	1.8	4.7	1.9	2.6	**6.7**
*cp127*	*rr1*	e	reg	A	A	1.0	1.0	11.2	**355.5**	188.4	115.5	1.1	1.2	82.0	**1325.8**	819.3	204.5	1.7	4.7	1.8	2.7	**6.7**
*cp128*	*rr2a*		reg	B	A	1.1	1.1	13.6	450.2	737.8	**839.2**	1.0	1.0	42.7	1227.7	**1848.7**	1432.5	1.5	6.1	2.5	3.5	**10.9**
*cp129/130*		e	reg	A	A	0.9	0.9	5.0	**138.8**	109.7	61.2	0.7	0.9	22.4	**584.6**	382.8	117.1	1.5	**6.7**	1.6	3.3	5.8
*cp131*	*lef-8*	e	reg	B	D	1.0	0.9	1.4	**19.4**	15.6	11.2	1.0	1.1	3.8	**72.5**	57.6	34.1	1.5	6.5	1.8	2.7	**12.0**
*cp132*				A	A	1.1	1.4	48.1	**2168.7**	1370.3	677.4	0.9	1.0	297.5	11140.9	7551.2	1129.5	1.7	6.2	5.2	2.7	**6.9**
*cp133*		e		B	D	0.9	0.9	2.2	34.1	**44.5**	41.8	0.8	0.7	2.9	81.4	**95.5**	49.3	1.5	3.3	1.3	3.1	**9.6**
*cp134*		l		B	A	1.0	0.9	2.3	99.9	**108.2**	98.9	1.1	1.3	17.0	378.8	**402.8**	145.9	1.7	4.9	1.7	2.6	**6.8**
*cp135*		l		A	A	1.1	1.2	18.7	**696.0**	520.6	343.0	1.1	1.1	85.8	**3089.3**	2105.5	551.2	1.5	4.8	2.5	2.7	**9.7**
*cp136*		l		D	D	1.0	0.9	2.2	127.7	230.9	**338.7**	1.1	1.2	16.1	610.0	**889.9**	732.2	1.7	4.7	1.7	2.6	**7.0**
*cp137*	*lef-10*	e, l	aux	B	C	0.9	1.0	1.8	2.5	**3.7**	3.6	1.0	0.9	1.1	3.8	**4.9**	2.9	1.5	3.2	1.1	3.6	**16.9**
cp138	*vp1054*	e	struc	B	D	1.1	1.0	4.5	124.3	**158.6**	145.8	1.2	1.3	15.4	351.1	**405.0**	256.1	1.7	9.1	1.9	3.9	**16.2**
*cp139*		e		D	C	1.0	1.0	1.5	5.4	9.6	**11.8**	1.0	1.0	1.3	14.9	**26.1**	17.0	1.4	5.3	1.5	3.1	**10.7**
*cp140*	*fgf-3*	e	aux	A	A	1.0	1.0	42.0	**1976.1**	1541.5	979.7	1.1	1.4	370.8	10208.7	7765.3	2401.2	1.7	4.8	3.4	2.7	**6.8**
*cp141*	*egt*	e	aux	A	A	1.0	1.0	41.9	**1007.4**	487.7	281.4	1.1	1.4	483.2	**4999.3**	2872.0	742.4	1.7	5.2	2.3	2.7	**6.6**
*cp142*		e		B	C	1.0	0.9	1.2	11.3	**13.5**	11.8	1.2	1.4	1.8	60.3	**74.8**	44.3	1.7	4.2	1.7	2.6	**6.7**

Since the same cDNA samples were used for the microarray and qPCR analysis, both data sets were used for the validation of the microarray results by comparing the transcription levels of the marker genes *ie-1*, *pe38*, *lef-8*, *f-protein*, *vp39* and *gran*. All replicates were combined by calculating the geometric mean of transcript levels and then normalized by dividing by the transcript values of the uninfected control (Table 2). To compare the two analyses the 2^-ΔΔCq^ values of the qPCR and the normalized geometric means from the microarray analyses were directly compared (Fig. S1). In summary, for all six genes the transcription levels measured by qPCR were generally higher than the levels determined by microarray analysis. According to the qPCR approach, the abundance of gene transcripts increased exponentially from 12 to 72 h p.i. followed by a further, though slight, increase up to 120 h p.i. (*ie-1*, *vp39*, *f-protein* and *gran*) and even a decrease (*lef-8* and *pe38*) (Figs 2a and S1). The changes in expression patterns derived from the microarray analysis were similar but were not as extensive as by qPCR.

### k-mean clustering of viral genes

**Fig. 4.**
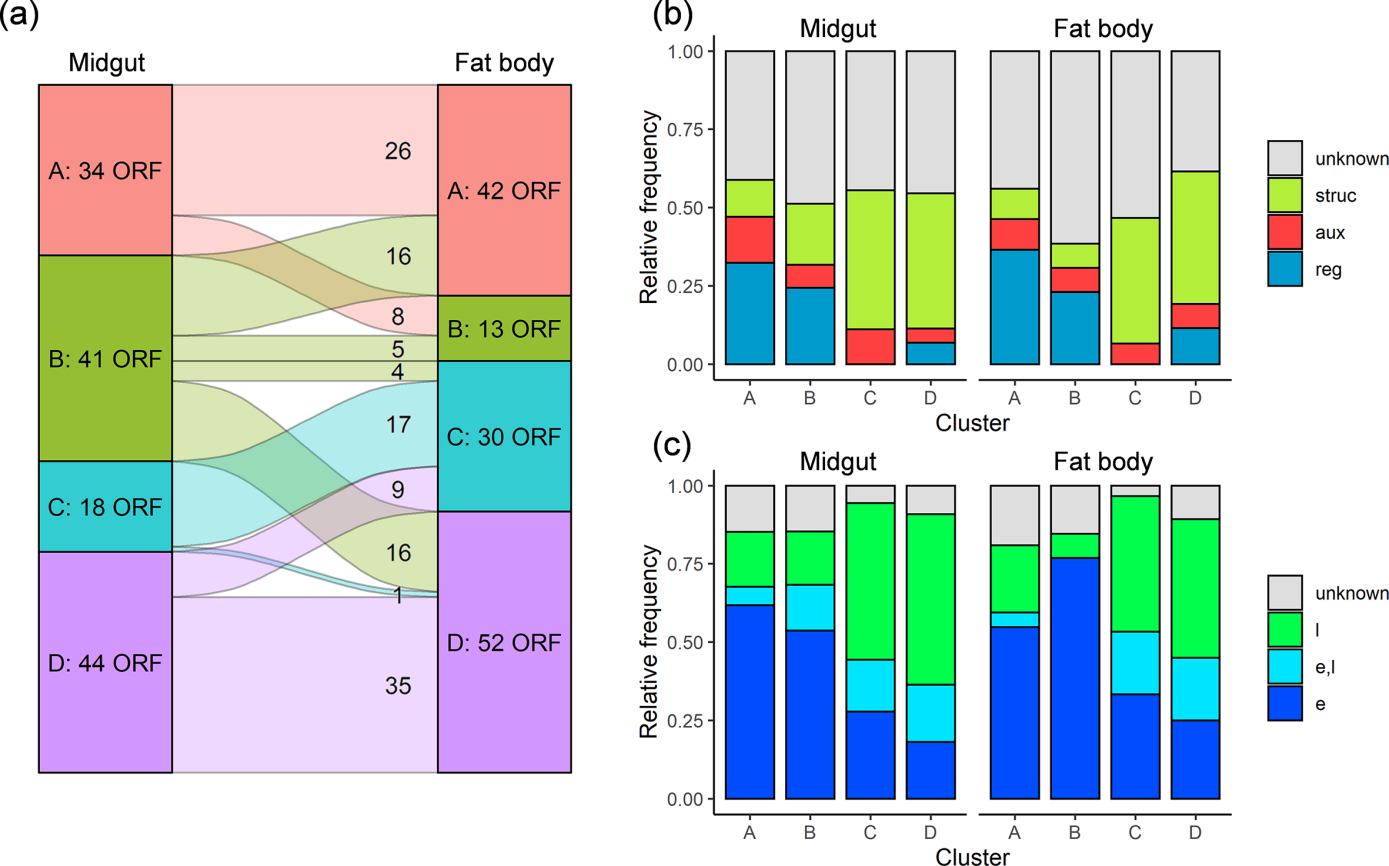
(a) Distribution of ORFs of CpS/CpGV-M within the four k-mean clusters A to D of midgut and fat body tissue. (b) Frequency of ORFs with regulatory (reg), auxiliary (aux), structural (struc) and unknown function. (c) Frequency of ORFs with an early (e), late (l) and both early and late (e, l) promoter motifs.

To analyse potential patterns and differences in the time course of the 137 ORFs, the gene expression values from the midgut and fat body tissue were divided into four groups (clusters A to D) by k-mean clustering (Fig. 4a, Table 2). The clustering into four groups was based on a step-wise testing of k groups until visual examination provided the most homogenous clustering. Clusters A and B were characterized by an increase in gene expression between 24 to 72 h p.i. followed by a trend in decreasing (cluster A) or constant (cluster B) levels of expression (Fig. S2). Clusters C and D include genes for which the expression values started to increase at 48 h p.i. but remained below 100-fold for cluster C, whereas they were much higher in cluster D. For the expression in the fat body, the selection of four clusters was also supported and was performed accordingly (Fig. S2).

The ORF compositions of the clusters were not identical in the midgut and fat body (Table 2) and several genes switched between the clusters (Fig. 4a). For the midgut, the clusters contained 34 ORFs (cluster A), 41 ORFs (B), 18 ORFs (C) and 44 ORFs (D) while in the fat body they were assigned to 42 ORFs (A), 13 ORFs (B), 30 ORFs (C) and 52 ORFs (D) (Fig. 4a). A total of 83 of the 137 ORFs (60.6 %) were assigned to the same clusters in the midgut and fat body (Fig. 4a). Cluster B was identified as the most unstable one, as it included 41 and 13 ORFs in the midgut and fat body, respectively. Only five ORFs, namely *cp27*, *cp72*, *lef-1 (cp74*), *lef-6 (cp80*) and *cp82a*, remained in cluster B, whereas 16 ORFs each from midgut cluster B were assigned to clusters A and D in the fat body. In addition, four ORFs from midgut cluster B were reclassified to cluster C in the fat body (Fig. 4a, Table 2).

### Gene function and promoters assigned to clusters

After the grouping of genes into four clusters, their composition was analysed concerning the assigned gene function and promoter motif(s). The genes were classified based on homology to well characterized alphabaculovirus ORFs and canonical promoter sequences upstream of the start codon of each predicted ORF (Table 2, Fig. 4b, c). A total of 75 of the 137 analysed ORFs (54.7%) of CpGV-M could be functionally assigned (Table 2). Altogether, 39 (28.5 %), 12 (8.8 %) and 24 (17.5 %) of these ORFs with a proposed function were assigned to encode structural, auxiliary and regulatory proteins, respectively. Another 62 ORFs (45.3 %) were of unknown function (Table 2). After the grouping into the clusters A to D of the midgut and the fat body, the relative proportion of ORFs with unknown function remained rather equally distributed over all clusters in both tissues. Clusters A and B contained mainly regulatory and auxiliary genes, whereas clusters C and D were dominated by genes encoding structural proteins (Fig. 4b). Interestingly, no regulatory genes were assigned to cluster C, neither in the midgut nor in the fat body.

For 121 (88.3 %) ORFs presence of an early (40.9 %) or late (33.6 %) or of both early and late (13.9 %) promoter motifs was found; for the remaining ORFs no promoter motif could be identified (11.7 %) (Table 2, Fig. 4b, c). For both tissues, ORFs with early promoters were found in mostly clusters A and B, whereas late promoters belonged predominantly to ORFs of clusters C and D. ORFs with both early and late promoters were also predominant in clusters C and D. Since regulatory and auxiliary genes have typically early promoters whereas structural proteins are transcribed late or very late in infection, it is not surprising that a certain correlation between the prevalence of gene and promoter classes can be noted (Fig. 4a, b)

### Differential expression of CpGV genes in midgut and fat body

**Fig. 5.**
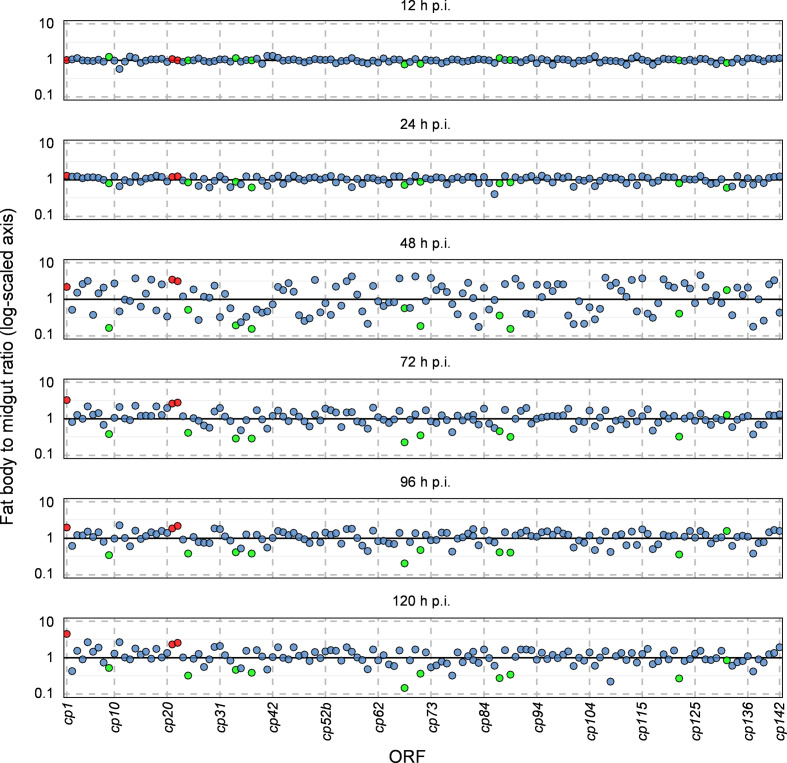
Expression ratio of CpGV-M transcribed genes in midgut and fat body of susceptible CpS larvae. Genes with higher transcription in the midgut between 48 to 120 h p.i. [*ac145* (*cp9*), *pe38* (*cp24*), *cp34*, *cp36b*, *cp67*, *cp70*, *lef-5* (*cp87*), *cp89*, *cp122* and *lef-10* (*cp137*)] are marked in green. Three genes were transcribed dominantly in the fat body between 48 to 120 h p.i. [*gran* (*cp1*), *orf17L* (*cp21*) and *orf17R* (*cp22*)] are marked in red.

Gene-expression values in the midgut and fat body in both tissues were compared directly to each other. First, the expression values of each time point (Table 2) were normalized by its respective geometric mean. Then, the normalized expression values at each time point in the fat body were divided by the normalized expression values of the midgut of the same time point resulting in quotients for each gene at 12, 24, 48, 72, 96 and 120 h p.i. (Fig. 5). The initial normalization by the geometric means helped to normalize the overall higher abundance of gene transcripts in the fat body (Table 2). The obtained quotients fluctuated around the value 1, whereas transcription ratios >1 indicated higher expression levels in the fat body and a value <1 indicated relatively higher expression levels in the midgut. Since the overall expression of genes was quite low within the first 24 h p.i., the values varied only between 0.6 and 1.3 (12 h p.i.) and 0.4 and 1.3 (24 h p.i.). After 48 h p.i. the differences in the transcription ratio (0.15 to 4.6) of both tissues were most significant and a generally strong scattering of the values was observed. At 96 h p.i. and 120 h p.i. the transcription ratios were between 0.2 to 2.3 and 0.15 to 4.5 (Fig. 5). While at 120 h p.i. the genes *gran* (4.5×) and *cp67* (0.15×) in particular marked the maxima of differential expression levels in the midgut and fat body. By the direct comparison of the midgut and fat body transcriptional levels, a group of 30 genes was discovered (48 to 120 h p.i.) with a higher transcript abundance in the midgut than in the fat body. Ten of these genes *ac145* (*cp9*), *cp34*, *cp36b*, *cp67*, *cp70*, *lef-5* (*cp87*), *cp89*, *cp122* and *lef-10* (*cp137*) showed 2.5- (ratio 0.4) to 6.7-fold (0.15) higher transcription in the midgut than in the fat body. Especially *p6.9* (*cp86*) was found to have higher transcriptional rates in the midgut than in the fat body throughout the entire microarray study. From 48 h p.i. onwards, 32 genes appeared to be more highly expressed in the fat body than in the midgut, most of these encoded structural proteins. The *gran* (*cp1*), *orf17L* (*cp21*) and *orf17R* (*cp22*) were the top three of this group (Fig. 5).

### Evaluation of viral gene transcripts of CpGV-M in resistant CpRR1 larvae

Finally, the transcription levels of CpGV-M in midguts of infected CpRR1 larvae was compared to that in CpS larvae. As mentioned above, the initial RT-qPCR analyses had suggested that several pooled cDNA samples from infected CpRR1 larvae were contaminated with cDNAs from single larvae with a successful CpGV-M infection. Therefore, these samples were ignored, and the analyses was reduced to three replicates at 12 h, two replicates at 24 h, three replicates at 48 h, two replicates at 72 h, no sample from 96 h and one replicate at 120 h (Figs 2, 3c and S5).

In midgut tissues of CpRR1 larvae infected with CpGV-M the level of gene transcription varied between 1.7- (*cp122*) to 62.2-fold (*ac108*, *cp56*) (Table 2). The second highest expressed gene was *orf34a* (*cp42*) followed by *pep/p34* (*cp23*) with a 26.4- and 21.0-fold increased expression. For 44 ORFs the levels of gene expression were between 10- to 20-fold and for 90 ORFs below 10-fold increased expression levels. For 132 out of 137 ORFs the maximum abundance of transcripts was reached at 120 h p.i. 4 ORFs, namely *cp129/130*, *vp91* (*cp101*), *pif-3* (*cp35*) and *cp122*, reached their maximum at 24 h p.i. and *pp31* (*cp57*) reached its highest transcription rate at 72 h p.i., which was comparable to its peaks in the midguts of CpS larvae (Table 2). The transcriptional activity of all genes was highly reduced and no differentiation of the expression patterns could be achieved by k-mean clustering (Fig. S6), indicating that global transcription and onset of infection of CpGV-M was blocked in CpRR1 larvae.

## Discussion

Deciphering the molecular mechanisms of CpGV infection and gene cross-talk with its host is a challenging task that is impeded by the relatively large dsDNA genome of CpGV with about 140 ORFs [2] and only a recently sequenced *C. pomonella* genome [41] that was not available during the course of this study. In addition, hardly any of the CpGV ORFs are functionally studied and their potential function has been assigned because of their homology to baculovirus core genes or the intensively studied genes of AcMNPV, the best-studied member of the *Baculoviridae* family [40, 42]. The prediction of gene and promoter functions based on AcMNPV molecular biology was applied for baculoviruses from different genera and also led to the temporal classification of very early, early, late and very late transcribed baculovirus genes [43], which were also the focus of the current study.

As for most other baculoviruses, little is known about the actual CpGV transcript lengths and which neighbouring ORFs may have overlapping mRNAs. Most oligonucleotides used for the microarray study were located near the 3′ end of each ORF. If two ORFs are transcribed in the same direction and the 5′ transcript start site of the neighbouring downstream gene is located within the 3′ end of the adjacent upstream one, such oligonucleotides may not be able to differentiate between the two adjacent overlapping transcripts, hampering the correct quantification of transcripts of the target gene. Adjacent ORFs with the same reading directions, occurring only with about one third of the CpGV ORFs, would be necessary as a prerequisite for such a scenario. During the design of oligonucleotides the location of the identified early and late promoter motifs was taken into account to avoid such ambiguous location, however other parameters, such as oligonucleotide melting temperature, sequence complexity and homology, had also to be regarded for their design when using eArray software. Nevertheless, such unidentified overlaps may occur in very few cases but should not impact the overall information gained from the experiments.

To obtain a first impression on the temporal regulation of CpGV gene expression in susceptible and resistant CM larvae, the viral transcripts were analysed qualitatively and quantitatively by representatives of the four gene classes: *ie-1* and *pe38*, as two very early genes [44, 45], *lef-8* an early or delayed early gene [46], *f-protein* and *mcp* both late genes [47, 48] and *gran*, the very late gene [49]. Despite a missing synchronicity in infection of the larval midgut and fat body, the larval system was chosen preferably over a synchronized infection of cultured cells for several reasons. First, Cp14R is the only *C. pomonella* cell culture available supporting CpGV replication, and although permissive for CpGV, the infection is very slow and produces only low virus titres [30, 50]. Second, a resistant cell culture representing CpRR1 is missing entirely. Third, the larval system had the advantage of offering the possibility to distinguish between a primary infection of the midgut and the secondary infection of the fat body, therefore reflecting more adequately the conditions of an *in vivo* infection.

As found with RT-qPCR experiments, the transcription of the selected representative genes appeared to be highly similar and homogenous within the permissive treatments with CpS/CpGV-M as well as CpRR1/CpGV-I12 concerning the transcriptional increase and temporal pattern. Temporal differentiation of the gene classes was not possible by the qPCR analysis, which might be the consequence of the non-synchronous infection of cells and tissues. However, an overall increase in gene expression over time was clearly visible, indicating a spreading infection within the CpS and CpRR1 larvae infected with CpGV-M and CpGV-I12, respectively.

Interestingly, a distinct timely separation of the primary midgut and secondary fat body infection could not be observed, neither with the RT-qPCR experiment nor later in the microarray study. Either the time slot of transmission was missed, or the number of chosen time points was not frequent enough. Another reason might be the postulated bypass of the nucleus by the ODV-released nucleocapsids in the midgut [51–53]. In our experiments, the onset of transcription in the CpRR1/CpGV-I12 treatment appeared to be about 12 to 24 h p.i. earlier than in the permissive CpS/CpGV-M combination. At first glance, this finding seems to be in contradiction to the previous observation that CpRR1 larvae infected with CpGV-I12 died about 1 to 2 days later than CpS larvae infected with CpGV-M [54]. On the other hand, it is conceivable that the initiation of infection of CpRR1 with CpGV-I12 is accelerated, whereas the infection progress is delayed, resulting in an increased time to death.

In contrast to the susceptible treatments, the CpRR1 larvae fed with CpGV-M showed a highly heterogeneous response in the RT-qPCR studies, which was observable between biological replicates and between different time points. Any technical failure in the resistant treatment CpRR1/CpGV-M can be excluded because the susceptible treatments CpS/CpGV-M and CpRR1/CpGV-I12 resulted in highly consistent results. CpRR1 originated from progenies of single-pair crossing experiments and is considered to be genetically highly homogenous [23], though recent bioassay studies demonstrated that some individuals of CpRR1 still succumb to CpGV-M infection [27–29], suggesting that not all individuals of CpRR1 have the same expression of resistance. It is therefore assumed that some of the time points and replicates of the resistant treatment CpRR1/CpGV-M contained single susceptible CpRR1 larvae productively infected with CpGV-M and thus contributing to the observed qPCR patterns. This assumption is supported by the fact that there is a biologically inexplicable variability between independent samples from different time points and replicates but a strong correlation between midgut and fat body collected from the same larvae. The latter clearly underlines that the quality of RNA isolation itself was highly reliable.

Since there was no other functional explanation for why a subsequent time point has a greatly reduced or no transcriptional activity than a previous one with high gene expression, this observation was interpreted as resulting from some individual CpRR1 larvae, which were susceptible to CpGV-M and contaminated the pooled RNA samples. In this case, some replicates and time points contained signals from progressive infections with CpGV-M. But even in those samples, the overall transcription levels was generally 10- to 20-times lower than in the CpS/CpGV-M and CpRR1/CpGV-I12 susceptible treatments, strongly suggesting that a single susceptible CpRR1 larvae in the pool could have caused the observed patterns. A leaky resistance of CpRR1 to CpGV-M was previously reported, in which 5–10 % of CpRR1 individuals were susceptible [16, 25].

For that reason, these possibly contaminated CpRR1/CpGV-M cDNA samples were excluded from the microarray analyses. In those samples which were considered to include only resistant CpRR1 larvae, low levels of transcription were noted by PCR and in the microarray analyses, suggesting a limited onset of CpGV-M infection in resistant larvae. This finding corroborates previous observations that CpGV-M may enter midgut cells but infection and virus replication are highly compromised [30].

In contrast, susceptible CpS larvae infected with CpGV-M showed rather structured expression patterns of genes which were represented by four groups of k-mean clusters. Although the expression patterns might have been partly obscured by the asynchronous infection of midgut and fat body cells, at the early stage of the larval infection regulatory genes and genes with early promoters were expressed, which changed at later times to expression of structural genes with late promoters, such as *gran* (*cp1*; cluster D), a highly expressed gene the transcripts of which are required during the very late stage of infection for the formation of OBs. Another important structural gene is *vp39* (*cp96*; cluster A) coding for VP39, the major capsid protein, that is required for the formation of newly synthesized nucleocapsids. Its expression peaked early, underlining the importance and spread of new nucleocapsids facilitating secondary infection. With the identification of four clusters, the present study for CpGV corroborates previous findings from AcMNPV and transcription expression levels in *Trichoplusia ni* cells. There, a grouping also in four clusters led to a similar observation: a temporal transition from mainly regulatory genes and early promoters to structural genes with late promoters [32].

The expression of *pe38* (*cp24*; cluster A), the candidate viral factor associated with type I resistance, was a particular focus of this study [29]. According to our results in the qPCR and microarray analyses, its expression at the early stage of viral infection was confirmed. The onset of early virus gene transcription in resistant host larvae was further noted by the transcription of *pp31* (*cp57*). PP31 is described as a non-essential protein associated to the virogenic stroma and effecting the transcription levels of other viral genes [55]. In midgut tissues of *Mamestra configurata* infected with Mamestra configurata nucleopolyhedrovirus A, the expression level of *pp31* ranked under the top seven most expressed genes [56]. In addition, transcripts of *p6.9*, a gene encoding for a DNA-binding protein, were most abundant in the same tissue [56] whereas *p6.9* (*cp86*) of CpGV-M was determined to be the second highest expressed gene and always more abundant in the midgut than in the fat body. Both genes belonged to cluster A with peaks at 72 h p.i. indicating their importance at the early stage of infection. The genes *pp31* (*cp57*), *p6.9* (*cp86*), *dbp* (*cp81*), *cp132, fgf-3* (*cp140*) and *orf36L* (*cp44*) were also identified as the highest expressed genes in an RNAseq analyses of CM infected with CpGV-M, corroborating the high quality of the microarray analyses (Xi Yu *et al*. unpublished).

Another important observation was the discovery of two groups of genes that were expressed dominantly either in the midgut [*ac145* (*cp9*), *pe38* (*cp24*), *cp34*, *cp36b*, *cp67*, *cp70*, *lef-5* (*cp87*), *cp89*, *cp122* and *lef-10* (*cp137*)] or in the fat body [*gran* (*cp1*), *orf17L* (*cp21*) and *orf17R* (*cp22*)] of infected *C. pomonella* larvae. Since their discovery was based on normalized data, this finding could not be explained by the later stage of infection with a generally higher abundance of transcripts in fat body tissue. It rather indicated a tissue-specific gene expression pattern of CpGV.

In summary, the *in vivo* microarray studies of susceptible CpS larvae infected with CpGV led to the identification of viral genes into four clusters differing in function and promoter composition reflecting the temporal cascade of baculovirus gene expression. Onset of gene activity was measured within 12 h p.i. in the midgut and 24 to 48 h in the fat body. The investigations further allow the conclusion that CpGV-M is able to enter cells and the nucleus of resistant CpRR1 larvae, otherwise no viral gene transcripts would have been detected.

## Supplementary Data

Supplementary material 1
