# Supplementary material for: Transcriptome of Cydia pomonella granulovirus in susceptible and type I resistant codling moth larvae
Source: J Gen Virol. 2021 Feb 24;102(3):001566. doi: 10.1099/jgv.0.001566 (PMC8515866; doi:10.1099/jgv.0.001566)
Supplement: Supplementary material 1 [file jgv-102-1566-s001.pdf]

Supplementary Material

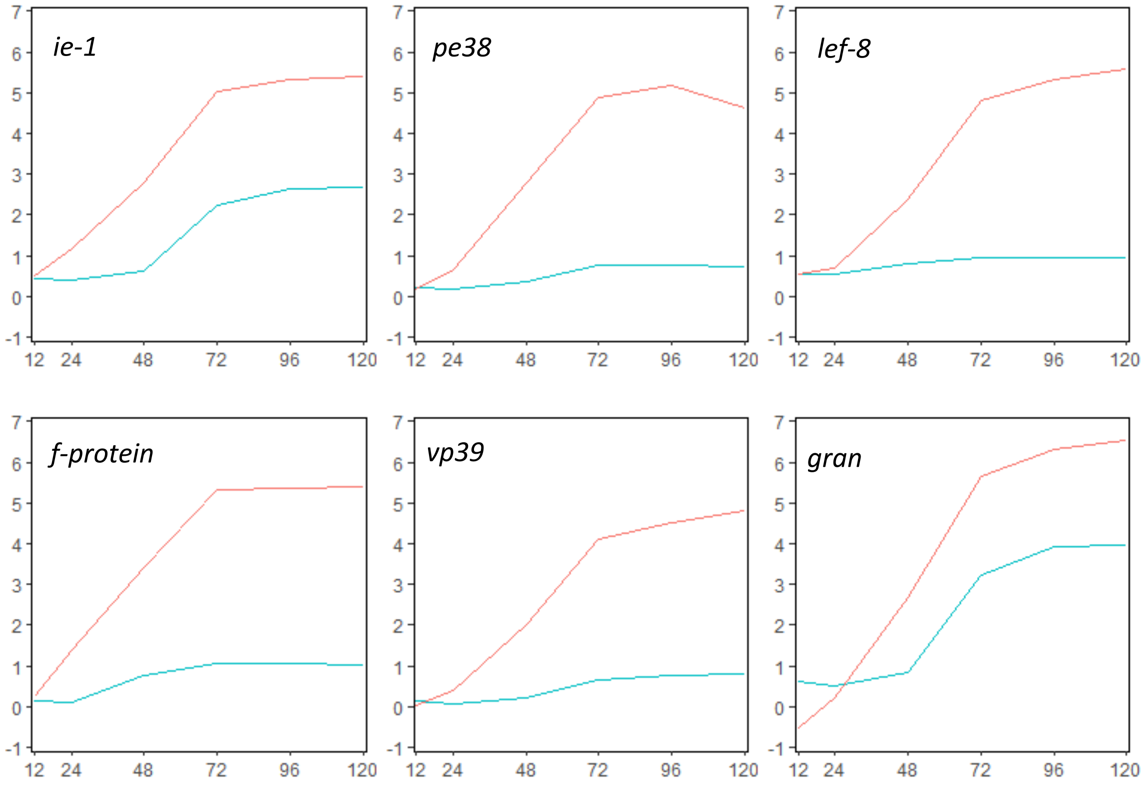

Figure S1: Log2-fold transcription levels of *ie-1*, *pe38*, *lef-8*, *f-protein*, *vp39* and *gran* as measured in CpGV-M infected midgut tissues of CpS larvae. The blue and red curve represent the microarray and qPCR-based data, respectively.

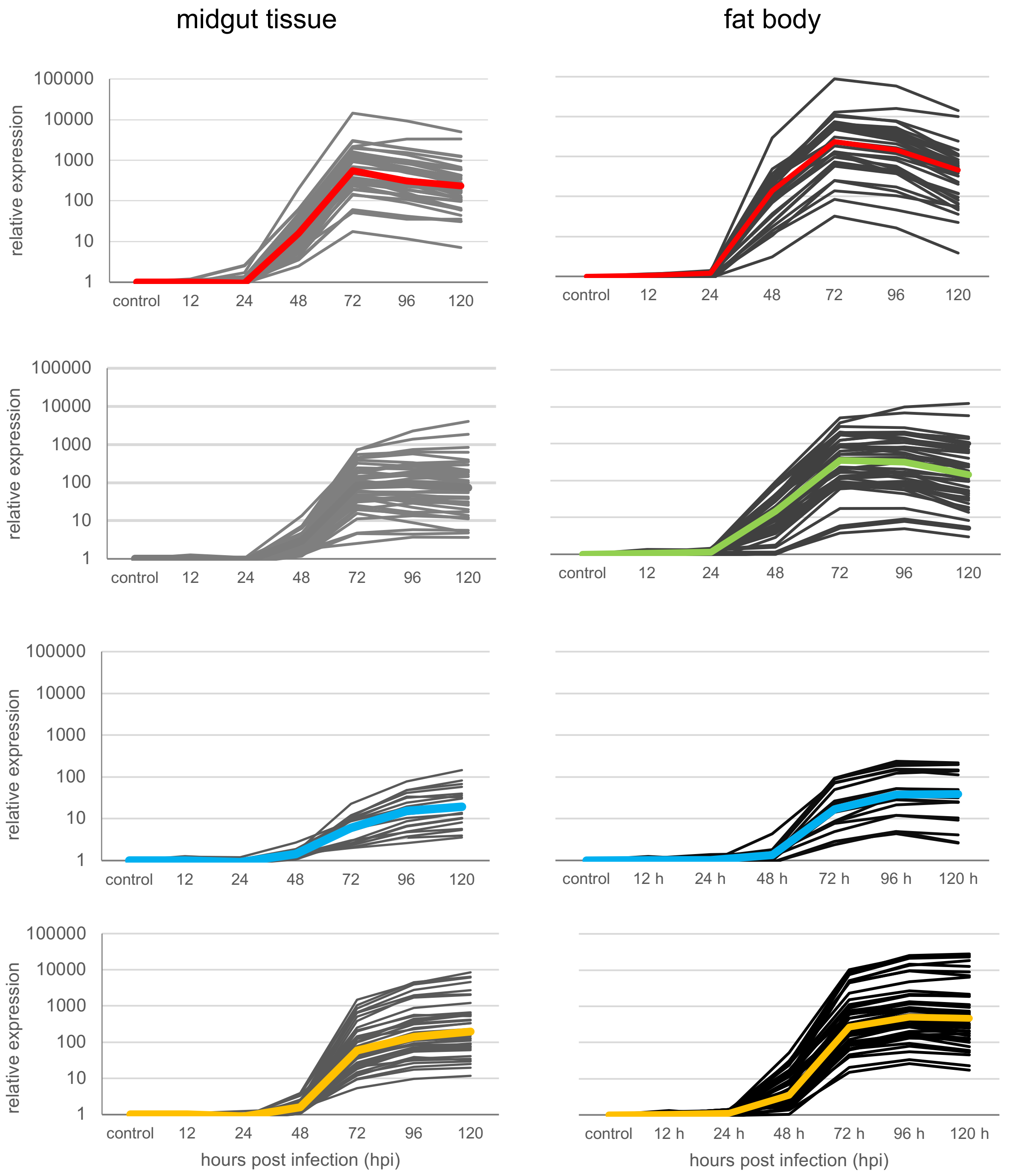

Figure S2: Relative abundance CpGV-M transcripts in CpS at 12, 24, 48, 72, 96 and 120 hours post infection (hpi) as measured by microarray analysis. Transcripts were grouped into clusters A (1st row), B (2nd row), C (3rd row) and D (4th row) for the midgut (left column) and fat body (right column) tissue based on profiles of temporal transcription.

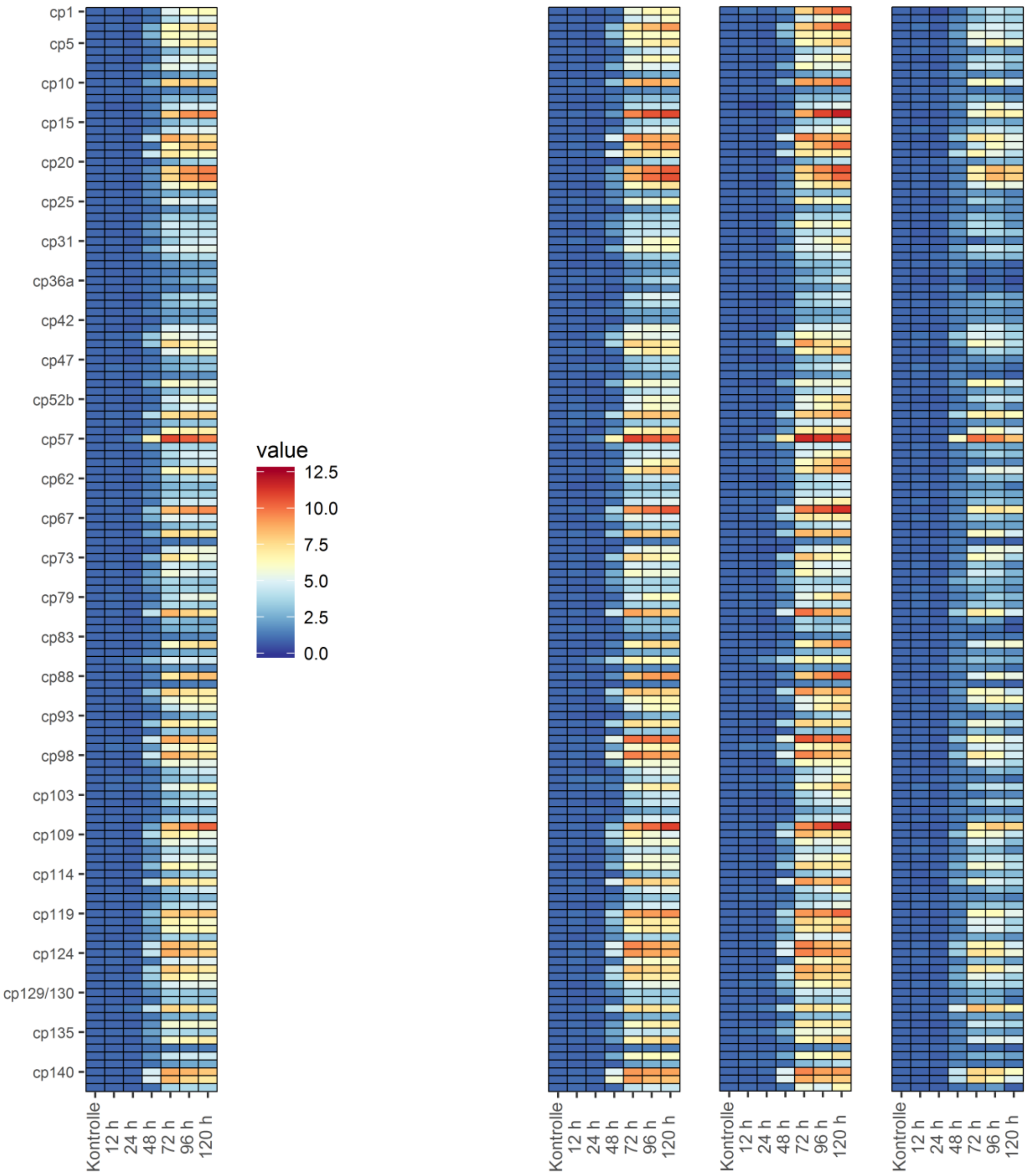

Figure S3: Heatmap of the temporal gene transcription within midgut tissue of the CpS/CpGV-M treatment. Values based on geometric means of Table 2 but log_2 transformed ranging from >0 (blue) to <12.5 (red) log_2-fold change. Geometric mean is shown on the left. The three replicates are shown independently on the right. Open reading frames are listed to the left of the first heatmap and are numbered vertically from cp1 to cp142 (Table 2). ORF cp40, cp72, cp75, cp106 and cp143 were not included (see text for further information).

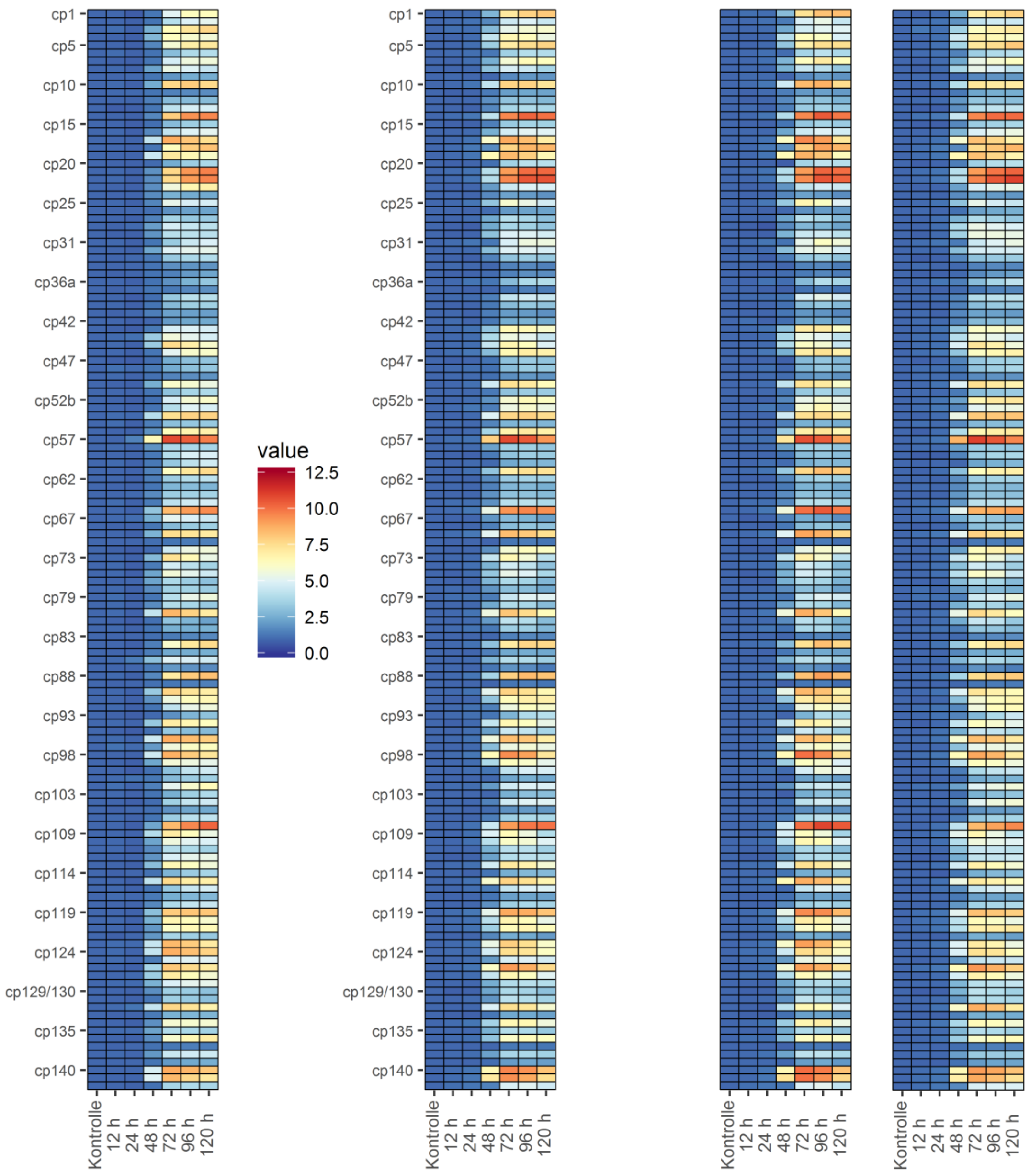

Figure S4: Heatmap of the temporal gene transcription within fat body tissue of the CpS/CpGV-M treatment. Values based on geometric means of Table 2 but log_2 transformed ranging from >0 (blue) to <12.5 (red) log_2-fold change. Geometric mean is shown on the left. The three replicates are shown independently on the right. Open reading frames are numbered vertically from cp1 to cp142 (Table 2). ORF cp40, cp72, cp75, cp106 and cp143 were not included (see text for further information).

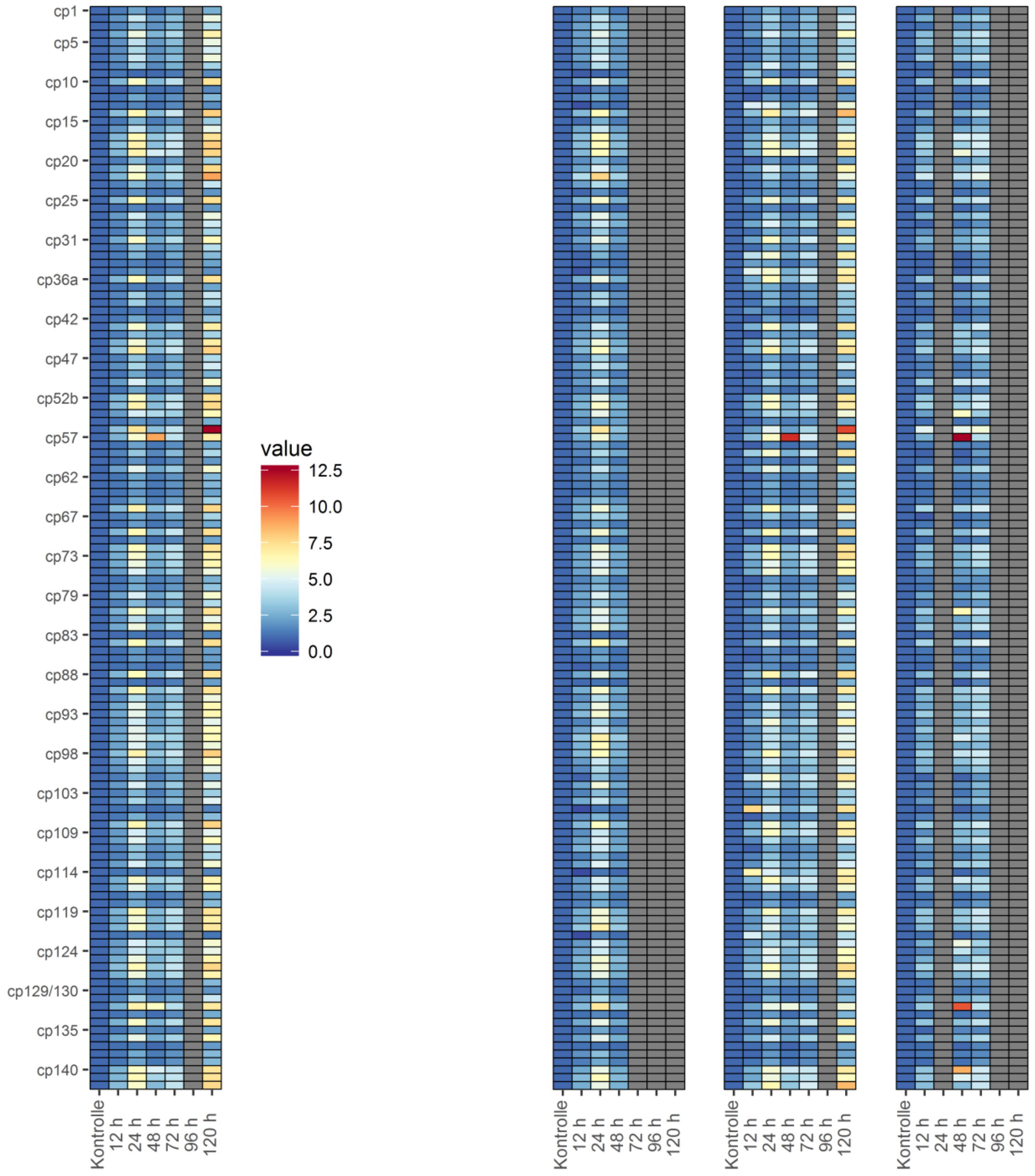

Figure S5: Heatmap of the temporal gene transcription within midgut tissue of the CpRR1/CpGV-M treatment. Values based on geometric means of Table 2 but $\log_2$ transformed ranging from >0 (blue) to <12.5 (red) $\log_2$-fold change. Missing transcription data is marked in grey. Geometric mean is shown on the left. The three replicates are shown independently on the right. Open reading frames are numbered vertically from cp1 to cp142 (Table 2). ORF cp40, cp72, cp75, cp106 and cp143 were not included (see text for further information).

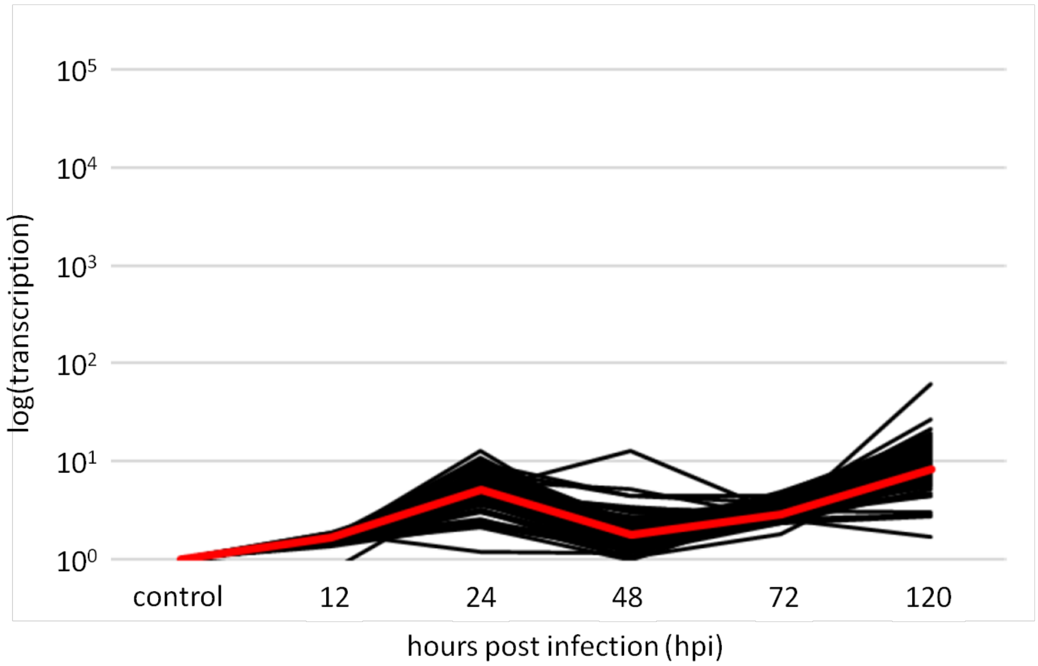

Figure S6: Viral gene transcription of CpGV-M in the midgut tissue of CpRR1. The red line represents the median transcription of all genes.

Table S1: List of 137 60mers that were used for the microarray based gene expression analysis of CpGV-M.

ORF no.	Annotation	Position and orientation	Probe ID	60mer sequence
1	*gran*	658 > 717	CUST_1_PI427296273	ATTGAAGAGATCATGATTGAGGTGTCGCTCTTGTTCAAGATCAAGGAGTTTGCACCCGAC
2	*cp2*	811 < 752	CUST_2_PI427296273	AAACGCATTGCCGAACAGTTTGTGGACGATGATGATTACTTTGACAAATTGTCAGTTCAC
3	*pk1*	1956 > 2015	CUST_3_PI427296273	TACTCTAAACCGCTACCCACCATTGAACACGTGTCCAAAAAGGCCAACGATTTTGTTAGG
4	*cp4*	2235 < 2176	CUST_4_PI427296273	ATAGAGGCTTGTCACGCATTCGATAGGGCTAGTGCTATTTTAATACAGAATATTAAATTG
5	*cp5*	2912 > 2971	CUST_5_PI427296273	CCGTTGTTGGAGGGTACAAAAAATGCTTACGACAAATTTGTGAGCGAAAACGGAAAATGA
6	*cp6*	3239 > 3298	CUST_6_PI427296273	CACATACAACAAGGTGGCGAGACGAGTCCGACTTTATGGGCCAGAATCGGAAATTTTTAA
7	*ie-1*	3466 < 3407	CUST_7_PI427296273	GAGACGTTCGTTTGATTTGCTGCAAAACGATTGTAGCAAACTCAAATTAAGTTACAAGAA
8	*cp8 (Ac146)*	5416 > 5475	CUST_8_PI427296273	AGAGCGCTCAACTCCAAAGTGAAGAACGTAAATTACTTACATGGTCTGTGTGTGGCTGAG
9	*cp9*	5736 < 5677	CUST_9_PI427296273	TTTGAATGTGACTCTTACTTCAAGTGTCCGGAGGGTATAAAGTTCTATTGCGACGTGGGC
10	*chitinase*	6087 < 6028	CUST_10_PI427296273	TTTTGTCCGTTCAAAAACGCCATCATTTATGCCATCAATTCTGTGTGCAATTCAGTATGA
11	*chathepsin*	8777 > 8836	CUST_11_PI427296273	GTGTTGCTGGTGGGATACGGGGTGAAAAACGATGTGCCATACTGGATTTTGAAAAACAGT
12	*cp12*	9189 > 9248	CUST_12_PI427296273	GCAAAAAGAATTATCAAGCGGCTTATCAAAAAATTCCAACCAAACCAAGCGCCGATATGA
13	*gp37*	9380 < 9321	CUST_13_PI427296273	GGCGGAGAGGGTTTCTATAATTGTGTGGATGTTGTTCACAAGCAAACGAAAAGTGATTTG
14	*odv-e18*	10308 < 10249	CUST_14_PI427296273	TCATTATCATATTGCTGGTGTTGTTGTTCAACATGAGCAGTGGTAGCGATAGCTCGGGCT
15	*p49*	10540 < 10481	CUST_15_PI427296273	GCGCCCATATACATTGTGGTGCGTGTGGACGACAATTTAATAGTAAGACATAATTTAATA
16	*cp16*	12207 < 12148	CUST_16_PI427296273	AACCTGGACGAGCAGCAACAAAAAGTGTGCTCCATGATTGAAAGCGCGTTTAGTAGTGAA
17	*iap3*	13630 > 13689	CUST_17_PI427296273	GTAGATAAGTGTCCGATGTGTCGCAAAATTGTGACCAGTGTATTGAAGGTGTATTTTTCT
18	*odv-e56*	13820 < 13761	CUST_18_PI427296273	GTTGGTGATAGGCGGGATATTATTACTAACCTTTATTGGGTTTGTTATATTCAAAGTGGT
19	*orf15R*	15339 > 15398	CUST_19_PI427296273	GAAAACAGGCTGAACGCTTTGCGTAACGATTATTTGAAGAAAATTGTAGATAGGCTGTAG
20	*orf16L*	15525 < 15466	CUST_20_PI427296273	AGGAGGTTAACTGTAAAATAAACAGAATCTACGACATTCTATTGAACAGGGAGATCACAA
21	*orf17L*	17813 > 17872	CUST_21_PI427296273	ACGTCTCAATGATTACACGGACGACTTTGAGAAGTTACTGAAAAATGTTCAAGTAGTGAA
22	*orf17R*	17817 > 18876	CUST_3_PI427397103	CTCAATGATTACACGGACGACTTTGAGAAGTTACTGAAAAATGTTCAAGTAGTGAAAAAG
23	*cp23*	18355 > 18425	CUST_22_PI427296273	ATCAGCAAAACAGAGGATGACGTGCTGAATCTATTGAATGAACCGGTGCCTACACCAGTT
24	*pe38*	18634 < 18575	CUST_23_PI427296273	GTACATTGTTAAAAGTTTGACTGATTCGTTGCGTCGTGCCACCAAAAAACCCATTAAATA
25	*cp25*	20293 < 20234	CUST_24_PI427296273	GCACCTCTTACTTTAGTCAGTTACAGCAGTTTCATGAGCTTGCAAATTTTGATTAATTGT
26	*cp26*	21525 > 21311	CUST_25_PI427296273	AAACAAAAACTGGTAAAGCTGGCGGTTGGTGTACTGGGGACGGATACACAAGAAATTTTC
27	*cp27*	20644 < 20585	CUST_26_PI427296273	AAGAGGATGAAAAACAACAGCTGCGCAACATTCTGATGGTGTTTGAGCGGAGTGACCTTA
28	*cp28/29*	29989 > 24048	CUST_27_PI427296273	TAGGGAGATTATTAAACTTATTGAGGGTTCGTTTGAGGAACACGGTTTTTGTACTACAGA
30	*cp30*	25091 > 25150	CUST_28_PI427296273	GTTGAAACCGTGGCTAGGGTGTGTGAAAAGAAAAATTACAAAATTGTGTACAGTAACATA

ORF no.	Annotation	Position and orientation	Probe ID	60mer sequence
31	*f-protein*	26996 > 27055	CUST_29_PI427296273	TATTAATTTTGGTAATGTTTGTGCTCTACACGAAACGTGTTTGCTGCGGAGATGGCTGTG
32	*cp32*	28587 > 28646	CUST_30_PI427296273	CTTGTACACCAAGTACCAGGAGATGTTGACGCGTCTAAATTATTTTGAGAACAAATTAAA
33	*cp33*	28799 < 28740	CUST_31_PI427296273	AGTTTCAACAAATTGTTAAAGGGGTCGGTGGGTTTATTGGAGGTATTGCTACAGTGGAGT
34	*cp34*	29723 < 29664	CUST_32_PI427296273	CCCTCAGATGTACTCGTCTCGCTCGATAACAAGCGTGTACCCTTTTTCAACATTTGGTAA
35	*pif-3*	30262 > 30321	CUST_33_PI427296273	TTTCTTATTTTGATGGTCATTGTGGGCATGGTGGTGTTAGTGAATGGGTGGTTGCGTACA
36	*cp36a*	31057 < 30998	CUST_34_PI427296273	TGTTGTACAGGGCAGAGGCTGCAAGTATTTGCAGCAAAGCAGGTGTATTATGCACATTAG
36	*cp36b*	30992 < 30933	CUST_35_PI427296273	TAGTAGTTGTCGTCGTTGTACACCCAATCGGCGAGGTCGTCGTTGGTTATGTAGTAAAAA
37	*odv-e66*	31267 < 31208	CUST_36_PI427296273	TACAACAGCGGAGTGTACACCTTGACGAATAACAATTATCTACAATTTTGGTTTGACTAC
39	*cp39*	33736 > 33795	CUST_37_PI427296273	AGATTTAACCACCAACTGTCACCAGAATTAGGGTCGGGGTACATGTGGAACACACATTTT
41	*lef-2*	34773 > 34832	CUST_38_PI427296273	TTTTTACAAAATGGATTCCAAGTGTGTTAGTCAGGTGGATGGGTTGGTGGCGCGTTTAAC
42	*orf35a*	35104 > 35163	CUST_39_PI427296273	CAACTCTGAGATGACCAGGTGCTTAGTAAACAAATGTAGGAATACACACAAAATATATTG
43	*cp43*	35283 < 35224	CUST_40_PI427296273	GGAGCAGAATAGGGTAATTAATTTTGCAAATGACTACGTGCACATGTTTGTTGATGAATA
44	*orf36L*	35695 < 35636	CUST_41_PI427296273	GTCGAACAACGACCACAATTATTTCTCCATAAGCACCAAATTTATAAACACGTACCTGTG
45	*cp45*	36389 < 36330	CUST_42_PI427296273	TATCGCCAATTGTAACAACACCCTGGATGATGTCATATGCGACGGTGACTATTTGCTGAG
46	*mp-nase*	37217 < 37158	CUST_43_PI427296273	TTTGTTTGTGGACCACCATTTGTGGTTCGAGTACACGGACACCACACTAGATAGAGTTAG
47	*p13*	39124 > 39273	CUST_44_PI427296273	GGTGGGTGGAGAAAAAACATCTTTCTACATGGACACCTTTATTTGGAAATATTTCTACCA
48	*cp48 (pif-1)*	40304 > 40363	CUST_45_PI427296273	ACCGGAAATTTTCAATCAAAACACTGATTTCGCGTACACATTCACGCTGCACGGAGTGGT
49	*cp49*	40512 < 40453	CUST_46_PI427296273	CACCAGATGATAAATCGACTATCCATAATCACATTACTAGCCATGATCAAAGCCACTTAA
50	*cp50/51*	44028 > 44087	CUST_48_PI427296273	GCTGATGATGATAATATGGATGAAGATGATGAGGAACAGTCCATTGTAAACAATAGATTG
52	*cp52 a (Ac107)*	44324 < 44265	CUST_47_PI427296273	AAACATCGAGCCGGTGGATAATAAGAAAAGGTTGTCGTTGAGCGAAAGGGTGTCTTTGGT
53	*cp52b (Ac110)*	44936 > 44935	CUST_50_PI427296273	TTGGTGTACGAACATAAGTTCATACCAGTGGTTTTGGGAAAGTACGTTAACAAGTTGTAA
54	*v-ubi*	45165 < 45106	CUST_51_PI427296273	AGAGCACGCTGCACTTGGTATTAAGATTGAGAGGTGGCGAAGACTGGATATGCATTTGTA
55	*cp55*	46436 > 46495	CUST_52_PI427296273	TACAACGAACTAGGCACCAGAAAAGCGTATCTTCACGCGCCCCTCTACAACTATTTTTTG
56	*cp56*	46643 > 46702	CUST_53_PI427296273	CGTTTAGAGGAACAATTTGAATTGTTGAGGTTGAGGGAGGCGTATATAAAGAGTAATAGA
57	*pp31/39K*	46886 < 46827	CUST_54_PI427296273	TTTTTGGAGAGACACAATTTTATGAAGAGAGATGACGCTGAAGCGAAGGGCGTGTTGAAA
58	*lef-11*	47572 < 47513	CUST_55_PI427296273	CCTACCGACCACCATTAGAGACGAGTACACCTACTGCCTGAATAGAAACAACCAAAATGA
59	*sod*	48004 < 47945	CUST_56_PI427296273	ACAACATGATCAGTTTATATGGCGCGTACAGCATATTGGGTCGTAGTTTGGTGGTGCACA
60	*p74*	48696 < 48637	CUST_57_PI427296273	CTCTTGGTGCTCGTCACACTATGTATCACATTGTTCATTCTACCCTCTGTCCAATATTAC
61	*cp61*	50962 < 50903	CUST_58_PI427296273	GTCTGGTTTTGCGCCAAGATTTTGCCGAGAAAAAGTTTTTAGCTAAAATTTCTGGAAAAA

ORF no.	Annotation	Position and orientation	Probe ID	60mer sequence
62	*cp62*	51153 < 51094	CUST_59_PI427296273	AAGGATGAACCACACAAATCGACTGTAGCCGACCACCATCCAGAAATGGTTGTAGAACAT
63	*cp61 (bro)*	51856 > 51915	CUST_60_PI427296273	ATAAACTCTCTCCGAAAAGACAATGGCCTCAGTTTCAACCGAGACCGAGGCTGTTATTAC
64	*cp64*	53236 > 53295	CUST_61_PI427296273	AAATATTCTATGCTTAGTCGTAATGATCGTCGCCGTAAACGTAAATACGAACCCTGCCAC
65	*(Ac79)*	53503 < 53444	CUST_62_PI427296273	CGTATAGTTTCAGAACAACCTAAAATTCCTATAAGTCAGCCTACAGTGCGCAACCTATGA
66	*ptp2*	54000 > 54059	CUST_63_PI427296273	TTGCGGAACAGATGTCATTATGTGTTTCGCGAAAACAAGAAGTGTGTGGTAGTTTTTTAG
67	*cp67*	54193 < 54134	CUST_64_PI427296273	CCAGTGCGTCGTCGTAGCCGCTCTAGGTCCATACAATCCGACGACACTGTAGTCTTGTGA
68	*p47*	55642 > 55701	CUST_65_PI427296273	ACATGGAGTTGAAGGTGGATTTTATTAAAGGGAAACGCATCACCACCGGCACACATGATC
69	*cp69*	56373 > 56432	CUST_66_PI427296273	CTGGCACTTATGATTACTTTTTTCAATTAGATCTGATGTGGTATGTGGAGAGTGAGAGGT
70	*cp70*	56680 < 56621	CUST_67_PI427296273	GTGAACAAGTGTTTATGTGCATGAACAATAACGCCTCCTCCAACTACGCCACCAGAAATT
71	*p24*	57681 > 57740	CUST_68_PI427296273	GAGCGGGAAGTAATGTATGGTATTGAAAGTAATAATAGTGGTGGCAGTGAAAATGGTACT
73	*38.7kd*	58433 < 58374	CUST_69_PI427296273	ATTTTGACACATATTACTGATAACGTGGTGATAGCGGTGGGTGATTGTGAGAGTGTAAAG
74	*lef-1*	58979 < 58920	CUST_70_PI427296273	CGCCGTACAGTTATAATAGCAAGGGTCAAAAGTTTAGCTGTGATCATGTCCTACTTTTTG
75	*pif-2 (Ac106)*	61292 > 61351	CUST_49_PI427296273	TGCCACGGTACCACAATCACAATTCAATACATTAATACAACTTTTGGCCACTTATCCATA
76	*cp76*	61640 < 61581	CUST_71_PI427296273	ACACTACAAAGACTATTCCCTGTGGAGGAAACTGCTGATACTGATTGGTGTGGTGGAGTA
77	*cp77*	62382 < 62323	CUST_72_PI427296273	GGGCGACCAGAATCTGTACAACGAGGCGCGGCGCGACATTATGAAGCACTTAATAAAAAA
78	*cp78*	62650 < 62591	CUST_73_PI427296273	ATATATAACACACATCGTACACCATATCTTCATCATTACTGCACCGGGACCGCATACTAG
79	*(Ac150)*	63211 > 63270	CUST_74_PI427296273	TTGTCGTCGACTTGTTACAACGCAAACACACTGTACTCCATAGAGACCCAAGAGTGTGAA
80	*lef-6*	63391 < 63332	CUST_75_PI427296273	AACATATGCAGGAGATTGACGACAACGATGACGACAGCAGTATTTCGCCCGAAGACTATT
81	*dbp*	63752 < 63693	CUST_76_PI427296273	TCCATGTGGTACACACCAATCGTGTTCATCTATGTACGTAAACCGGGTGAGGATCAGTAA
82	*cp82a*	64651 < 64592	CUST_77_PI427296273	CAGCGATTGAATAAAAAGCAGATAGACGGCTGTGTACAGTACAGTAGGGACGACGATTAG
82	*cp82b*	65001 < 64942	CUST_78_PI427296273	TCAGTACTGTGTCAGTACACAACGTGAACGTTTGGAGCAAATAGCCACATTGGTGTTCCA
83	*p45*	66615 > 66674	CUST_79_PI427296273	AATTTGAAGAACGACGTGGGTAGGTTGATGCAGAAAAGTGTGCGTTTGGGTGTAATATAG
84	*p12*	66976 > 67035	CUST_80_PI427296273	GGTATAGTGAATAGCAAGTTTGTCATACAGGAGGATGCCAACTCAATGATCGTGGATTGA
85	*cp85 (odv-c42)*	68099 > 61158	CUST_81_PI427296273	AATTTTGTTGAAAAAGTGGCGGCCGGCGACATTAGCATCAAAAAACGCATCACCACCAAC
86	*p6.9*	68358 > 68417	CUST_82_PI427296273	CGTTCTCGATCACGCTCTCCATATCGTTCTCACTACCACCACATAAATCAGTACATATAA
87	*lef-5*	68787 < 68728	CUST_83_PI427296273	TACTCGATAAACTGGAAGAACAGTTTAAACAAAAAACGGGTGTTGGCGGGCAAGAGAAAG
88	*cp88 (Ac98)*	70042 > 70101	CUST_84_PI427296273	TGGCATTCGCGTATATTGCGGTACATTGTTCAGTATGATAACATGTTTAATAGGTGTTAA
89	*cp89 (Ac96)*	70329 < 70270	CUST_85_PI427296273	CAACTCAATAATTGATATTATTAACTATTTGATTAGAAGCGGCTCGGCACAGATAACGTG
90	*helicase*	74074 > 74133	CUST_86_PI427296273	GAAAAGTATTTTCAGACGTTTACGCCCAAATTCAAGGCTAATGTGGATGTTACTGTTTAA

ORF no.	Annotation	Position and orientation	Probe ID	60mer sequence
91	*odv-e25*	74309 < 74250	CUST_87_PI427296273	ACACACAAATCAACGACAGTGCCACGTTGTTTGTGAAAAATGAAAAAAGTTTTAGGTTAC
92	*cp92 (Ac93)*	75038 <74979	CUST_88_PI427296273	CAGCTGTTGGTCGAGTTGCAGAGCAAAAACTTTATCACTCTAATGAAAACTGTTAGTTTG
93	*cp93*	76197 > 76256	CUST_89_PI427296273	CCCATACAACATGATAGGGAAGAGTTGAACAATTTTCAAAGAATGGAGTGGAGCATATTG
94	*iap*	76386 <76327	CUST_90_PI427296273	GATAGACGGTGTCCCGTGTGTAGACAAAAAATTGTATTATTCACACCGGTGTTTTTGAAT
95	*lef-4*	77121 < 77062	CUST_91_PI427296273	TCGTGCAGCAAGATAAAGTGAAGGTTTTAAAAGAGAGACCGGACAGATTGATAAGTAATT
96	*vp39*	79202 > 79261	CUST_92_PI427296273	TTATGGAGTTTGACACGTGCACATTTACCTCAGAGGGTTTGACCGTGCCAAATTTGCACA
97	*odv-ec27*	80337 > 80396	CUST_93_PI427296273	ATCGTTGCCCAACGTAAACAATCTCATCATTGGACACTTTAGTTTTAACGTGGAGGCGGG
98	*ptp2*	81179 > 81238	CUST_94_PI427296273	TTGAAGGGAAAGTGTGTGTATAGTTATGTGGATAATAAGTTGGTGGTTAGAGTTGAATAG
99	*cp99*	81390 < 81331	CUST_95_PI427296273	TTGCGTACAACAAAGTTTACGAGGCAAGAGAGGTTTGGGATAAGGAGCGAAGAGAAAAAT
100	*cp100*	82812> 82871	CUST_96_PI427296273	GGTCACATCAACAGATTGCCTAGCAATTATAGGCGAGATCCGAGAGTTATGAAATTTTAA
101	*vp91*	82965 <82906	CUST_97_PI427296273	TTTGATTTGGCGCAGCGCAACGTTGGAGATTACTCGTGTTTGTTCACTGTTCCTGTTTAA
102	*tlp20*	85472 > 85531	CUST_98_PI427296273	CCCGCTACTAGTAAGACCTCGTTGTTTGATAAATACTATGTCGAGTCAAGAAGGAAGCAG
103	*cp103 (Ac81)*	86006 > 86065	CUST_99_PI427296273	CTTTGTCAACAATTACCTATTGATGGAGCCGCGCGTAGAGTACTGTAAGCATTACAAGAC
104	*gp41*	86910 > 86969	CUST_100_PI427296273	AAACGAGGCTTTGCGACGCGGTAAAATACAAGAGTTGAACATGAAGTATGCCAATTTGCG
105	*cp105 (Ac78)*	87226 > 87285	CUST_101_PI427296273	TATTTTGGCGGTGTTGTTGTTGGTGGGTTACTTTGTTTTGTGGCGGTACAATGACGACAC
107	*cp107 (Ac76)*	88703> 88762	CUST_102_PI427296273	CAGCACCTAAACGACGCCTTTGATGCATTGTTAAACAAAAACAATTCGTCCATAGAATAA
108	*cp108 (Ac75)*	89220 > 89279	CUST_103_PI427296273	TTAAGAGTCATTTAATGAACACCATCGACGATCAGGATGGGTTTGCTAACATGAGTATTT
109	*cp109*	89477 < 89418	CUST_104_PI427296273	GTACCGCCTTCTTGGTTAAAAGTTGTTAAACACGATGATGTAGATGACATCATGTTATTG
110	*cp110*	90411 < 90352	CUST_105_PI427296273	CACCAGCTGGACGATGACACGCAAGCTGAATTGATTTATTGTGTGGAGGATGAAAATTAA
111	*dnapol*	90908 < 90849	CUST_106_PI427296273	CTGGAGTTAGTGGTGGAAAAACAAAGTGCGTCACGCAAGCGACCTTCAAAGCAAAATTAG
112	*desmoplakin*	96041 > 96100	CUST_107_PI427296273	GAAAGGGTGGTATCCTGCCTATTAAAAACATTTCATTCGTCAAACCTAATGTTAGGAAAT
113	*lef-3*	96372 < 96313	CUST_108_PI427296273	AACATTGTGTCGATGGTTATTGATGACGAGGAGAAGACCTCAACATTGATGGCGGCTTAA
114	*cp114 (Ac68)*	97661 > 97720	CUST_109_PI427296273	ATAGTGTCGGGTCTTATATTAATTATGTTATCGTATTTGGTGGTGGAAGGGATGAGCAAA
115	*cp115*	98273 > 98332	CUST_110_PI427296273	GCCTAAGGAGGCGTGGTTTTTTAGTCAGTGTTACATTTGTAATTATTGTTTCACCAATAA
116	*iap-5*	99279 > 99356	CUST_111_PI427296273	GGTTAAAATTCCGTACAATAAGCTGAATCTGGTGGAACATGAGCGGATACCGAATCGAGT
117	*lef-9*	100733 > 100792	CUST_112_PI427296273	ATTTGCTACAAACAAAACACGCTTAGTTTGGGCAGCATCGCCCACCTACCCGAACAATTT
118	*fp25k*	101288 > 101347	CUST_113_PI427296273	GGTCGCGCTCACAAATTCACGTTATCAAAAACGAAACCGACATTGATTACATCAAAAGTT
119	*cp119*	101851 > 101910	CUST_114_PI427296273	CAATTATTGTGTAGGGTGTAGAAGAATTTTGTTTGATGTATTGGATGTTGAGGAGGAGGT
120	*dnaligase*	102082 < 102023	CUST_115_PI427296273	CAACAACAGTTTTAGTTATCCGTATCTGTACGAGTTTTTCGTCATGGACAACATTAAGAA

ORF no.	Annotation	Position and orientation	Probe ID	60mer sequence
121	*cp121*	104137 > 104196	CUST_116_PI427296273	ACACGGTGAAAAAGAGTTTTTCGCAAGATTCGAGTATTTGGTACACCACCAAGTCGGGTG
122	*cp122*	104448 > 104507	CUST_117_PI427296273	CTAGTCATCTTGTTGGTTATATTTGTCCTGTTATTGGTGCCGTCTATTTTACTAGCTATA
123	*fgf*	104718 < 104659	CUST_118_PI427296273	GCAGTAGCAGTTTGGGTAACACGCTTACGATTAATATACCACACTTATTTTTGTTCTTAA
124	*cp124*	106130 > 106189	CUST_119_PI427296273	ACGAGTTTCCCAACACTAAATATGTGACTGTGAGCGGTTTGCAGAGTTTGGCGCGATTAT
125	*alk-exo*	107465 > 107524	CUST_120_PI427296273	TTTTCGATCGCATAAATAATTTGAACACGGTGGTGACAACGCTGTCTTCAGTCGAACGCC
126	*helicase-2*	108869 > 108928	CUST_121_PI427296273	GGTGTGAAGGATATGAGCTTTATTACCGAGGTGTATAACAGTATGGAAAAATGGTGTTAG
127	*rr1*	107076 < 109017	CUST_122_PI427296273	TATGAACAGGTGAAACGGGCTCGTTCAAAGTTTGGCGGATGTGATGGGTGTACATTGTAA
128	*rr2a*	111983 > 112042	CUST_123_PI427296273	AGTTCAATCTGATGGAGAGCACGTCCACGGTGTACAATGCCAGCAACAGCATTTATTATC
129	*cp129/130*	112251 < 112192	CUST_124_PI427296273	GTGGTCTGGCGTGAACGAGTTTTTATTTGTCAATAACGCTTGTCGGGAAAAATATAACAA
131	*lef-8*	113257 < 113198	CUST_125_PI427296273	GGACAATACAATGTGTTCCAAACATTGTTGCACTGCAACAATATCAAAGTGGTGAACTAA
132	*cp132*	116194 > 116253	CUST_126_PI427296273	AATTGTGTTGAACAATGAGCATATAACCAGCGTGTTTGGTAAGCTGCGGAGGGATGGAGA
133	*cp133*	116423 < 116364	CUST_127_PI427296273	CTGCAAATAGTGGAGGTGTTAATTGTTATAGCGGTGGCTTTGCATAAAATAAAACAGTGA
134	*cp134 (Ac53)*	116881 > 116940	CUST_128_PI427296273	ACTTTAAAATCGACCACAAGCGGTTACCAGGATGTGGAATTTCCGTTGGGATTGACATTG
135	*cp135*	117071 < 117012	CUST_129_PI427296273	CTTAGAGCACCCGCACCAACAATACCTGAACCCGTGTTTGTTATAGAGAACAGGTTTTAA
136	*cp136*	118207 < 118148	CUST_130_PI427296273	ACGGAGCCGTTGTTAGATTTGGACAATATAAGAGGTGACCAGTTTGTGGTTGTACACTAA
137	*lef-10*	118435 > 118494	CUST_131_PI427296273	TACACCACAACAATGAACTGCGCAGAACTTGTTACGGCACTATCGATTGCTGTCTACCAA
138	*vp1054*	119386 > 119445	CUST_132_PI427296273	CTGCGCGAATGGGCCATTTTCATCAACGGTTTTAAAACGCACTTTTTTCCCACACCGTAA
139	*cp139*	119430 < 119371	CUST_133_PI427296273	AAAGTGCGTTTTAAAACCGTTGATGAAAATGGCCCATTCGCGCAGCTGCTCTTGTCGTTT
140	*cp140*	120740 > 120799	CUST_134_PI427296273	ACGGTGACAATAGTTGTTAAGTTTAAGCGAAGATCGTTTCGGTTAACCAATAAAGTGTAG
141	*egt*	120942 < 120883	CUST_135_PI427296273	GGTGTTGAGAAACGGCAGTTTGATGTATGTGTTTAATGGCAATAAATATTGGAGTAAATG
142	*cp142*	122498 > 122557	CUST_136_PI427296273	CGGTTACAGTTTCATATTGAATTTGAACGGTCATGCAAGATTTCAGGACCCAGTTTTTGA
